# Deciphering the mechanism by which the yeast *Phaffia rhodozyma* responds adaptively to environmental, nutritional, and genetic cues

**DOI:** 10.1093/jimb/kuab048

**Published:** 2021-07-24

**Authors:** Luis B Flores-Cotera, Cipriano Chávez-Cabrera, Anahi Martínez-Cárdenas, Sergio Sánchez, Oscar Ulises García-Flores

**Affiliations:** Department of Biotechnology and Bioengineering, Cinvestav-IPN, Av. Instituto Politécnico Nacional 2508, Col. San Pedro Zacatenco, México city 07360, México; Department of Biotechnology and Bioengineering, Cinvestav-IPN, Av. Instituto Politécnico Nacional 2508, Col. San Pedro Zacatenco, México city 07360, México; Department of Biotechnology and Bioengineering, Cinvestav-IPN, Av. Instituto Politécnico Nacional 2508, Col. San Pedro Zacatenco, México city 07360, México; Department of Molecular Biology and Biotechnology, Instituto de Investigaciones Biomédicas, Universidad Nacional Autónoma de México, México city 04510, México; Department of Biotechnology and Bioengineering, Cinvestav-IPN, Av. Instituto Politécnico Nacional 2508, Col. San Pedro Zacatenco, México city 07360, México

**Keywords:** *Xanthophyllomyces*, Redox signaling, NADH/NAD^+^

## Abstract

*Phaffia rhodozyma* is a basidiomycetous yeast that synthesizes astaxanthin (ASX), which is a powerful and highly valuable antioxidant carotenoid pigment. *P. rhodozyma* cells accrue ASX and gain an intense red-pink coloration when faced with stressful conditions such as nutrient limitations (e.g., nitrogen or copper), the presence of toxic substances (e.g., antimycin A), or are affected by mutations in the genes that are involved in nitrogen metabolism or respiration. Since cellular accrual of ASX occurs under a wide variety of conditions, this yeast represents a valuable model for studying the growth conditions that entail oxidative stress for yeast cells. Recently, we proposed that ASX synthesis can be largely induced by conditions that lead to reduction–oxidation (redox) imbalances, particularly the state of the NADH/NAD^+^ couple together with an oxidative environment. In this work, we review the multiple known conditions that elicit ASX synthesis expanding on the data that we formerly examined. When considered alongside the Mitchell's chemiosmotic hypothesis, the study served to rationalize the induction of ASX synthesis and other adaptive cellular processes under a much broader set of conditions. Our aim was to propose an underlying mechanism that explains how a broad range of divergent conditions converge to induce ASX synthesis in *P. rhodozyma*. The mechanism that links the induction of ASX synthesis with the occurrence of NADH/NAD^+^ imbalances may help in understanding how other organisms detect any of a broad array of stimuli or gene mutations, and then adaptively respond to activate numerous compensatory cellular processes.

## Introduction

*Phaffia rhodozyma* (sexual state *Xanthophyllomyces dendrorhous*) is unusual heterobasidiomycetous yeast as it is the only yeast or fungus capable of synthesizing astaxanthin (ASX), a highly valuable carotenoid pigment with powerful antioxidant activity, and because other carotenoid-containing yeasts are generally strictly respiratory (Andrewes et al., 1976; Dose et al., 2016; Gassel et al., 2013). Astaxanthin safeguards *P. rhodozyma* cells against oxidative damage from reactive oxygen species (ROS) such as superoxide (O_2_^•–^), hydrogen peroxide (H_2_O_2_), and singlet oxygen (^1^O_2_), regardless of whether those result from a natural environment, or are otherwise generated through intracellular metabolism. This yeast also possesses a range of genes that are involved in its antioxidant defense, and are mostly shared by all eukaryotes, including a mitochondrial manganese-dependent superoxide dismutase (Mn-SOD) and low catalase activity as compared with *Saccharomyces cerevisiae*. The yeast can either lack or possesses low cytosolic superoxide dismutase activity (Cu/Zn SOD, SOD1), consequently, the yeast depends primarily on ASX for its antioxidant defense (Johnson & An, 1991; Johnson & Schroeder, 1996; Martinez-Moya et al., 2011; Schroeder & Johnson, 1993, 1995b; Zhang et al., 2020). Wolf et al. (2010) reported that ASX, at nM concentrations effectively maintains functional mitochondria under oxidative challenge.

Currently, the mechanism involved in the regulation of ASX synthesis by *P. rhodozyma* remains poorly understood (Alcaíno et al., 2014; Cordova et al., 2016; Gutierrez et al., 2019). However, it has been acknowledged that diverse nutritional and environmental conditions can trigger ASX biosynthesis (Johnson, 2003). As a result, *P. rhodozyma* cells accrue ASX and acquire intense red–pink coloration when faced with stressful conditions, which generally inhibit cell growth. Typical stressful conditions include; nutrient limitations (e.g., nitrogen or copper), the presence of toxic substances (e.g., antimycin A), or mutations in the genes involved in respiration or nitrogen metabolism, among others (Barbachano-Torres et al., 2014; Bhosale, 2004; Frengova & Beshkova, 2009; Johnson, 2003; Johnson & An, 1991; Rodríguez-Sáiz et al., 2010; Schmidt et al., 2011). Since the intracellular accumulation of ASX occurs in response to a wide variety of stimuli, this yeast represents an ideal model to study the growth conditions that entail oxidative stress on cells, *in vivo*. ASX cellular accrual occurs to some extent in proportion to the degree of the stress faced by the *P. rhodozyma* cells, therefore is a unique gauge to assess the relative importance of a given stressful condition because the conditions that activate ASX accrual must also promote the formation of intracellular ROS. It would be worthwhile to know the mechanism by which a wide range of different cues that lead to the ASX synthesis converge to activate the antioxidant response in *P. rhodozyma*.

The intracellular accumulation of ASX can be triggered by several different nutritional conditions, or the presence of respiratory inhibitors, or certain distinctive gene mutations. We recently reported that the onset of ASX synthesis appears to be elicited, in all cases, by common intracellular events, namely the emergence of reduction–oxidation (redox) imbalances, particularly of the NADH/NAD^+^ couple, in conjunction with the presence of oxygen (Martínez-Cárdenas et al., 2018). NAD^+^ (nicotinamide adenine dinucleotide) is a molecule that partakes in numerous biological reactions, and a pivotal cellular coenzyme in all living cells. In particular, NAD^+^ participates in the redox reactions of catabolism via the bidirectional conversion between the oxidized NAD^+^ and reduced NADH forms (Zhu et al., 2015). NADH is essential as a chemical energy intermediary and primary electron carrier in the cellular production of adenosine triphosphate (ATP). The oxidation of glucose throughout several stages of catabolism occurring in the cytoplasm (glycolysis) and mitochondria (tricarboxylic acid cycle or TCA cycle), is linked to the reduction of NAD^+^ to NADH. Therefore, the primary NADH sources are glycolysis and the TCA cycle. NADH plays a cornerstone role in cell metabolism because of its indispensable role as a biological source of hydride. In fermentative yeast cells, the oxidation of NADH to NAD^+^ predominantly occurs via two major routes: the cytosolic reduction of pyruvate to ethanol via alcohol dehydrogenase, or by feeding NADH electrons into the mitochondrial electron transport chain (mETC). In the latter instance, NADH oxidation coupled with the reduction of O_2_ to H_2_O has a Gibbs-free energy change of −220 kJ/mol (Aon et al., 2010; Brandon et al., 2006; Brune et al., 2000; Demasi et al., 2006; Møller, 2001). The energy released from the oxidation of NADH is conserved by driving the nonspontaneous reaction of joining adenosine diphosphate (ADP) with inorganic phosphate to generate ATP via mitochondrial ADP oxidative phosphorylation. The ATP produced by the cells is an immediate source of cellular energy that ultimately comes from the reducing power released from sugars, as a carbon source. The energy released by the reconversion of ATP to ADP is used to drive most cellular processes that require energy such as growth and cell division. The size of the NAD^+^ pool and the ratio of its reduced and oxidized forms shift in response to changes in the nutrient and environmental conditions, the ability to maintain a redox balance, which is the balance between NADH production and the oxidation of NADH, is therefore essential for all living cells (Green & Paget, 2004). In response to alterations in the NADH/NAD^+^ ratio, cells initiate new metabolic pathways and reconfigure their metabolism in a continued attempt to maintain the redox balance. Shifts in the reduction state of the NADH/NAD^+^ couple, depend dynamically on the balance between the production of NADH and its reoxidation to NAD^+^ within a subcellular structure (e.g., mitochondrion). Either excessive NADH production or impairment in the reoxidation of NADH may therefore result in a redox imbalance and a greater NADH/NAD^+^ ratio.

ASX synthesis by *P. rhodozyma* can be promoted by conditions that lead to redox imbalances such as (i) Impaired electron flow through the main mitochondrial respiratory chain (e.g., Cu^2^^+^ deficiency, or the presence of respiratory inhibitors such as antimycin A, among others). (ii) An excessive production of NADH, for example, via the assimilation of ethanol under oxidative conditions (Martínez-Cárdenas et al., 2018). One of our most important former findings was that *P. rhodozyma* cells repeatedly strive to preserve the global redox balance by inducing either ASX synthesis, alcoholic fermentation and/or other processes (Martínez-Cárdenas et al., 2018). In doing so, the cells protect themselves from the impact of conditions that favor the development of harsh intracellular oxidative stress. Indeed, the redox state is known to be a crucial determinant of cell functioning, and any considerable imbalance can cause severe cell damage or death (Dietz & Scheibe 2004).

In this study, we first briefly review the multiple events that are known to elicit the ASX synthesis in *P. rhodozyma*. In particular, we focus on and further expand on the range of data we recently examined (Martínez-Cárdenas et al., 2018). Second, we show that the mechanism that we formerly proposed (Martínez-Cárdenas et al., 2018), when taken in conjunction with Mitchell's chemiosmotic hypothesis (Mitchell, 1966), can serve to rationalize the induction of ASX synthesis, and other adaptive cellular processes, through events such as; deficiencies of nitrogen, phosphate or magnesium, or others, which we did not examine in our former publication (Martínez-Cárdenas et al., 2018). Our aim was to propose an underlying mechanism that explains how a broad range of divergent conditions converge to induce ASX synthesis in *P. rhodozyma*. Regardless of the stimulus that induces ASX synthesis, we now know that the induction occurs analogously following a common event in all cases, that is, the occurrence of a redox imbalance. Thus, the NADH/NAD^+^ pair can be considered a dynamic hub for cell signaling and coordination of the *P. rhodozyma* metabolism. The mechanism that links the induction of ASX synthesis with the emergence of NADH/NAD^+^ imbalances may well be regarded as a fundamental framework that may aid in understanding how different cell types detect any of a broad range of environmental stimuli or gene mutations, and then adaptively respond by activating numerous cellular processes.

## ASX Biosynthesis

Some carotenoids are found in the membranous areas of *P. rhodozyma* cells. Others, particularly non-oxygenated carotenoids such as β-carotene, are found in cytosolic oil droplets where they are associated with the activities of desaturases and cyclases (Johnson & Schroeder, 1996). This condition indicates that the early stages of carotenoid synthesis occur in the cytoplasm of *P. rhodozyma*, in a similar manner to the fatty acid synthesis in oleaginous yeasts. Mitochondrial citrate is the most important source of cytoplasmic acetyl coenzyme A (acetyl-CoA) for fatty acid synthesis in oleaginous yeasts (Evans et al., 1983). The same acetyl-CoA pool appears to be functional in the synthesis of both ASX and sterols in *P. rhodozyma* (Chávez-Cabrera et al., 2010; Flores-Cotera et al., 2001; Gomez et al., 2020; Gutierrez et al., 2019; Leiva et al., 2015; Miao et al., 2010, 2011). The biosynthetic pathway leading to ASX has been entirely described previously (Barredo et al., 2017; Schmidt et al., 2011). The earlier stages of ASX biosynthesis in *P. rhodozyma* include the mevalonate pathway, in which the key precursor acetyl-CoA is converted successively to hydroxymethylglutaryl-CoA (HMG-CoA) and mevalonate, and then transformed to isopentenyl pyrophosphate (IPP) (Andrewes et al., 1976; Johnson & An, 1991). IPP is an essential building block and common precursor for the endogenous synthesis of carotenoids, monoterpenes, sesquiterpenes, sterols, and gibberellins in fungi along with other compounds (Disch et al., 1998; Sandmann, 1994). The process begins when one IPP molecule and other of its isomer dimethylallyl pyrophosphate (DMAPP), each composed of five carbons (C5), undergoes condensation to form geranyl pyrophosphate (C10). The consecutive addition of IPP units generates farnesyl pyrophosphate (C15) and geranylgeranyl pyrophosphate (GGPP; C20). The condensation of two GGPP molecules gives phytoene (C40), the first and colorless carotenoid. A series of four desaturation steps then lead to lycopene, which then produces γ-carotene and next β-carotene (C40) via successive cyclization reactions at both ends. The final stages of the ASX biosynthetic pathway in *P. rhodozyma* are also well established. The formation of ASX requires the sequential oxidation of β-carotene; this process involves the addition of two oxo groups to carbons C4 and C4′ followed by the addition of two hydroxyl groups to carbons C3 and C3′ (Ojima et al., 2006). Four enzymes are required for ASX biosynthesis starting from IPP. The genes encoding the enzymes are GGPP synthase (*crtE*), phytoene synthase/lycopene cyclase (*crtYB*), phytoene desaturase (*crtI*), and ASX synthase (*asy*, formerly *crtS*), together with a cytochrome P450 monooxygenase that requires an electron donor for activity, that is, the product of *asr* (formerly *crtR*), a cytochrome P450 reductase (Alcaíno et al., 2008; Alvarez et al., 2006; Hoshino et al., 2000; Ojima et al., 2006; Schmidt et al., 2011; Verdoes, Krubasik, et al., 1999; Verdoes, Misawa, et al., 1999). The enzymes encoded by *crtYB* and *asy* are bi-functional; the first catalyzes the formation of phytoene and lycopene, the second has oxygenase and hydroxylase activity to transform β-carotene into ASX (Ojima et al., 2006; Schmidt et al., 2011). A monocyclic carotenoid pathway has also been reported in *P. rhodozyma* (An et al., 1999).

## Factors that encourage ASX Synthesis in *P. rhodozyma*

### Dissolved Oxygen Concentration [pO_2_]

Molecular oxygen is an essential substrate for mitochondrial respiration and oxidative phosphorylation in all aerobic cells. The accrual of ASX in *P. rhodozyma* cells typically occurs under aerobic conditions in which oxygen concentrations in the culture medium (pO_2_) are >20% of saturation with air (Chávez-Cabrera et al., 2010; Liu & Wu, 2008; Yamane et al., 1997a). The expression of carotenogenic genes has been consistently linked with the prevalence of O_2_ in the culture medium (Lodato et al., 2007). Furthermore, the oxygenated groups in ASX only form under oxidative conditions, whereas low pO_2_ levels or high concentrations of glucose lead to the accumulation of the precursor carotenoid β-carotene (Johnson & Lewis, 1979; Liu & Lee, 2000; Meyer & du Preez, 1994a). A strong linear relationship between carotenoid yield and oxygen transfer rate has also been reported (Liu & Wu, 2006b).

Large pO_2_ gradients exist toward the outside of cells as well as between different compartments within cells. Nevertheless, changes in the pO_2_ level of a given cell compartment depend on the balance between oxygen supply (by oxygen transfer from the cells milieu) and the oxygen consumed in the compartment. A higher O_2_ supply relative to O_2_ consumption, when saturation has not been achieved would increase the pO_2_ level in a culture medium. A high pO_2_ level or a rapid pO_2_ increase in a medium containing growing cells can cause oxidative stress in cells, leading to oxidation of macromolecules such as proteins, fatty acids and DNA (Konz et al., 1998). Cells that become overwhelmed by exposure to high concentrations of O_2_ or ROS are affected by non-specific damage to the cell biomolecules may promote the arrest of growth and ultimately cell death. ROS production is an inevitable consequence of aerobic life. Cells primarily produce O_2_^•–^, which is converted by SODs to H_2_O_2_, this latter is considered a central redox signaling molecule that targets specific redox sensors (Sies, 2018). Indeed it is well established from prior *in vivo* research with different cell types and isolated mitochondria from diverse sources that the O_2_^•–^/H_2_O_2_ generation rate and subsequent production of other ROS, positively depends on both the pO_2_ levels and the degree to which redox cofactors such as NADH/NAD^+^ are reduced (Fang & Beattie, 2003; Grivennikova et al., 2018; Hoffman & Brookes, 2009; Miñana et al., 2002; Murphy 2009; Quinlan et al., 2012, 2013). An increase in the generation of ROS usually occurs after levels of pO_2_ increase or reduced redox cofactors rise (e.g., NADH, QH_2_). The mechanism entails the electron transfer directly from a reduced cofactor to molecular oxygen to give O_2_^•–^ (Barros et al., 2004; Longo et al., 1999; Rigoulet et al., 2011).

Yeast cells that grow to the early stationary phase change their metabolic state from rapid proliferation (fermentation) to slow proliferation (respiration), which is concomitant with increasing levels of pO_2_. The increase in the pO_2_ levels of yeast cultures is a common event that promotes ASX accrual that typically occurs following the depletion of one of several key nutrients that are required for cell growth (e.g., glucose, nitrogen or phosphate). It should be emphasized that under some other conditions (see below), the onset of ASX accrual in *P. rhodozyma* cells is frequently associated with a decline in the mitochondrial respiratory function, which, simultaneously slows down the reduction of oxygen to water and the oxidation of NADH (Martínez-Cárdenas et al., 2018). Examples of such conditions include the presence of respiratory inhibitors (e.g., antimycin A), or gene mutations in cells that result in impaired electron flow through the mitochondrial electron transport chain (mETC). Conditions that trigger ASX synthesis are therefore apparently linked to increasing intracellular pO_2_ levels within the mitochondria and, presumably, with higher NADH/NAD^+^ ratios. This would be expected to result in increased ROS production and the need for further antioxidant protection.

Several studies have shown that exposure of *P. rhodozyma* cells to several ROS (e.g., O_2_^•–^, H_2_O_2_ or ^1^O_2_), or ROS generating compounds (e.g., TiO_2_) promotes ASX biosynthesis and increases both the carotenoid cell content and the relative amounts of xanthophylls present, that is, oxygenated carotenoids (An et al., 1996; Kim & Chang, 2006; Liu & Wu, 2006a; Schroeder & Johnson, 1995a; Zhang et al., 2019, 2020). For example, *P. rhodozyma* cells that are exposed to 10–20 mM H_2_O_2_ increased their intracellular ASX within 4 hr. Older yeast cells (aged 120 hr) were more tolerant to H_2_O_2_ toxicity than the younger cells (age 24 hr), which is likely due to their higher cellular ASX content (Liu & Wu, 2006a). Also, *P. rhodozyma* grown in presence of TiO_2_, at 500 mg/L, generates O_2_^•–^, H_2_O_2_ and HO•, and increases the cellular carotenoid content (Zhang et al., 2019, 2020). A proteomic analysis was performed to study the mechanism promoting ASX synthesis by *P. rhodozyma* under TiO_2_ stress (Zhang et al., 2020). The analysis revealed that TiO_2_ promotes ASX synthesis by a mechanism that might involve redox balance, translation of ribosomes, and ion transmembrane transport.

In fact, a pO_2_ > 20% plays a crucial role in triggering ASX biosynthesis, however, a high pO_2_ or prominent oxidative challenges can readily inhibit or arrest the growth of *P. rhodozyma* cells. Indeed, growth inhibition or arrest occurs repeatedly in parallel with the accrual of intracellular-ASX (An et al., 2001; Davies et al., 1995; Yamane et al., 1997a,b).

### Nitrogen Limitation

The nitrogen source and concentration of nitrogen in a culture medium play a crucial role in the production of microbial carotenoids (Braunwald et al., 2013). The accrual of intracellular ASX and the ASX/total pigment ratio in *P. rhodozyma* frequently increase when cells grown at low nitrogen concentrations or high initial carbon/nitrogen (C/N) ratios (Chávez-Cabrera et al., 2010; Flores-Cotera et al., 2001; Meyer & du Preez, 1994a; Pan et el., 2017; Vustin et al., 2004; Yamane et al., 1997a). Previous work has shown that the end concentrations of biomass, ASX and total carotenoids, all exhibit similar bell-shaped relationships with the initial concentration of ammonium sulfate (2.1–61 mM) in the culture medium (Flores-Cotera et al., 2001). The concentrations of biomass, ASX and total carotenoid pigments peaked (at 10.9 mg/ml, 2.4 μg/ml, and 3.9 μg/ml, respectively) when ammonium levels were between 12.9 and 28.6 mM. However, lower concentrations were observed at ammonium levels above 28.6 mM and below 12.9 mM. Contrasting, the ASX content in the cells increased from 140 to 302 μg/g when the initial ammonium concentration decreased from 61 to 2.1 mM. The increase in the cellular ASX accrual at the lowest ammonium level tested (2.1 mM) occurred jointly with a decrease in the cellular protein content and an increase in the cellular fatty acid content (Flores-Cotera et al., 2001). It is striking that this challenging condition, which strongly restricts yeast growth, still promotes the accrual of ASX by cells, which underscores the superlative priority of this antioxidant defense under such conditions.

When a nitrogen limitation occurs, the synthesis of new proteins and new cell biomass both slow down and ultimately become infeasible. The sharp decrease in biomass together with the disruption to the consumption of sugars at below 12.9 mM ammonium reported previously is evidence that cell replication was affected at such low concentrations, while the storage of lipids was observed to increase (Flores-Cotera et al., 2001; Li et al., 2015). When yeasts are grown in media in which only ammonium salts are the source of nitrogen, nitrogen availability has a significant effect on the amounts of all; carbon skeletons, ATP, and nicotinamide adenine dinucleotide phosphate (NADPH) that are funneled to amino acid and/or protein synthesis (Boer et al., 2010; Larsson et al., 1993, 1997; Warner, 1999). The global inhibition of protein synthesis is a common response to nitrogen deficiency, and some of the genes related to ribosomal proteins, tRNA synthetases, and initiation and elongation factors show decreased expression level under nitrogen depletion (Chávez-Cabrera et al., 2010; Torrent et al., 2018). The tricarboxylic acid cycle (TCA cycle) supplies the carbon skeletons that are required to synthesize structural biomolecules, as well as the reducing equivalents (e.g., NADH) for ATP synthesis (Flores et al., 2000). Ammonium exhaustion impairs growth and compulsorily reduces the carbon demand for protein and nucleotide synthesis, particularly α-ketoglutarate and oxaloacetate, which are mostly involved in ammonium assimilation. In addition, diminished protein synthesis results in a decline in the requirement for ATP, resulting in slower ATP turnover (Warner, 1999). An extensive uncoupling between anabolic energy requirements and catabolic energy production takes place when ATP is present in excess (Larsson et al., 1997). Therefore, ADP-shortfall reduces the synthesis of ATP by oxidative phosphorylation as an essential measure to maintain a balance between the production and consumption of cellular ATP. It is known that this coordination minimizes the production of ROS (Noctor & Foyer, 2000).

**Fig. 1:**
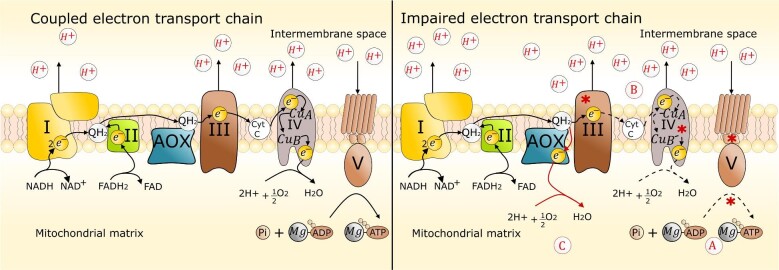
Coupled mitochondrial respiratory chain in which electron transfer from NADH to O_2_ via the multiprotein complexes I–IV, is coupled with the translocation of protons from the mitochondrial matrix to the intermembrane space to generate a proton-motive force (pmf). The pmf is used by the F_0_F_1_-ATP synthase (Complex V) to drive the combination of ADP with inorganic phosphate to make ATP. The electrons reaching complex IV are used to reduce O_2_ to water. The NADH/NAD^+^ couple is mainly in an oxidized state due to the rapid transfer of electrons to oxygen in the respiratory chain. In contrast, figure on the right shows several means (shown with red asterisks) by which the electron transport is impaired. (A) By impairing oxidative ADP-phosphorylation (e.g., under N deficiency, P deficiency, presence of inhibitors that affect the F_0_F_1_-ATP synthase function, and mutations that impair N metabolism). (B) By impairing electron flow through the mETC (e.g., copper deficiency, the presence of respiratory inhibitors such as antimycin A, or gene mutations impairing the electron flow through the mETC). The NADH/NAD^+^ ratio and pO_2_ are both increased relative to the coupled respiratory chain as a result of the slower electron transfer to oxygen. The latter conditions promote increased ROS production and consequently ASX synthesis. The dashed arrows signify a slower electron transport through the respiratory chain. (C) *P. rhodozyma* cells under copper limitation activate an alternative oxidative (AOX) denoted by the red arrows, which promotes the mitochondrial reoxidation of NADH, therefore the over reduction of the NADH/NAD^+^ couple is avoided and cells avert from being exposed to an excessive ROS production.

According to the chemiosmotic theory that was established in 1961 by Peter Mitchell, the electron flow from NADH and FADH_2_ to O_2_ that occurs via the multiprotein complexes of the mETC is coupled to the translocation of protons from the mitochondrial matrix to the intermembrane space (Berry et al., 2018; Mitchell, 1961; Mitchell, 1966). Proton translocation generates a proton-motive force (pmf) across the mitochondrial inner membrane, which is a form of potential energy that consist of charge (Δ*ψ*) and chemical (ΔpH) components that together drive ATP synthesis. The F_0_F_1_-ATP synthase allows H^+^ ions to diffuse back into the matrix and the free energy released is used to synthesize ATP from ADP and inorganic phosphate (Pi) (Fig. 1). Mitchell's chemiosmotic theory also established that the degree of coupling between the electron flow through the mETC and the oxidative phosphorylation of ADP to ATP varies. Accordingly, the pmf rises whenever ATP usage diminishes, exerting feedback control over the electron flow through the mETC. The respiratory apparatus must regulate the relative rates of NADH reoxidation (electron flow) through the mETC that regenerates NAD^+^, and ATP production via oxidative phosphorylation. In this way, the electron flow is diminished when ATP is abundant and the pmf increases (Brown, 1992; Mitchell, 1966). The mitochondrial oxidation of NADH together with reduction of O_2_ to water can therefore only proceed if sufficient ADP is present. This phenomenon of respiratory control is important, but is not the only mechanism by which NADH oxidation and ATP synthesis by oxidative phosphorylation are controlled. This avoids the derivation of constraints on the operation of respiratory energy transduction from imbalances in the availability of either of the two forms of assimilatory energy, ATP and NADH.

During aerobic growth, cells tend to maintain the NADH/NAD^+^ couple in an oxidized steady state (low NADH/NAD^+^ ratio), owing to the rapid transfer of respiratory electrons to oxygen, thereby ensuring high glycolytic fluxes (Zhao et al., 2016). However, under increasingly serious ammonium limitation, the electron flow through the mETC is theoretically constrained as a result of the restricted oxidative phosphorylation of ADP. A restricted electron flow would also be expected to affect the aerobic reoxidation of NADH leading to a reduced state of the NADH/NAD^+^ couple. Similarly, impaired electron flow slows down the reduction of oxygen to water, which leads to an increase in the pO_2_ levels within the culture medium. (Arnold & Kadenbach, 1997; Sluse & Jarmuszkiewicz, 1998). As mentioned previously, high pO_2_ levels together with a high degree of reduction of redox cofactors (i.e., high NADH/NAD^+^ ratio) are favorable conditions that predispose cell to greater O_2_^•–^/H_2_O_2_ generation (Ghyczy & Boros, 2001; Hoffman & Brookes, 2009; Miñana et al., 2002; Murphy, 2009; Quinlan et al., 2012, 2013). It is striking that cultures of *P. rhodozyma* cells initiate the intracellular accrual of ASX shortly after nitrogen limitation occurs, warnings of which are apparent in the increasing pO_2_ levels in the culture under these conditions (Chávez-Cabrera et al., 2010). These data and our interpretation are consistent with the theoretical prediction that ammonium deficiency positively supports greater ROS generation.

**Fig. 2:**
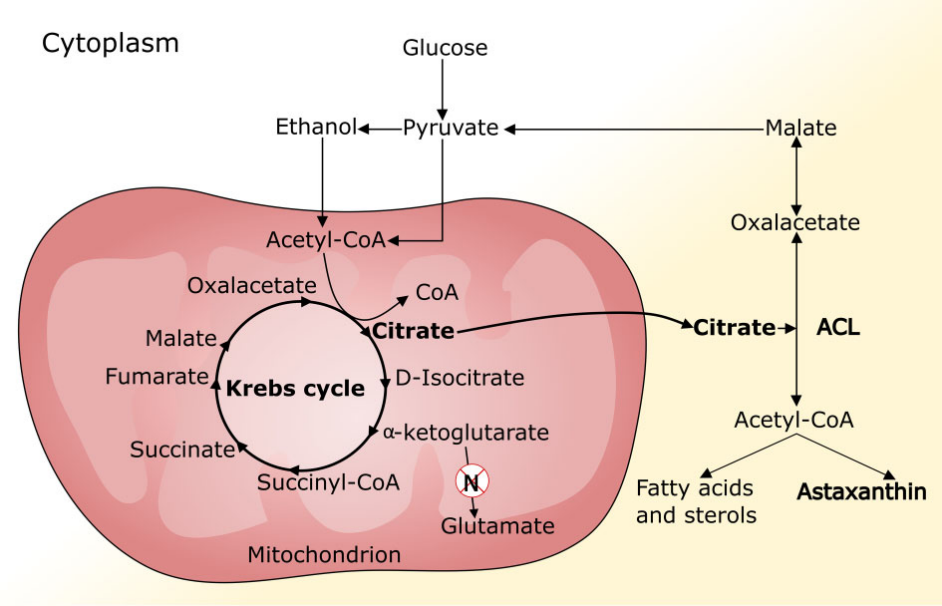
Pathway that supplies acetyl-CoA for fatty acid synthesis (Evans et al., 1983; Ratledge & Wynn, 2000) and ASX (Chavez-Cabrera et al., 2010) in oleaginous yeast shortly after occurring a nitrogen limitation. Nitrogen limitation promotes high cellular NADH and ATP that inhibit NAD^+^ isocitrate dehydrogenase. This elicits mitochondrial accumulation of intermediaries upstream of α-ketoglutarate in the TCA cycle, that is, citrate. Then citrate is exported from mitochondria to cytoplasm and cleaved by the enzyme ATP:citrate lyase (ACL) to give cytosolic acetyl-CoA and oxaloacetate. Citrate is considered the most important source of acetyl-CoA for the syntheses of fatty acid synthesis and ASX in yeasts grown under nitrogen-deficiency. *P. rhodozyma* possesses a citrate-malate shuttle for the translocation of citrate to cytosol.

The enzyme NAD^+^ isocitrate dehydrogenase (ICDH), which is involved in α-ketoglutarate formation, is commonly characterized by its susceptibility to inhibition by both NADH and ATP. Thus, high intracellular concentrations of NADH or ATP should reduce the activity of this enzyme, resulting in inhibition of the TCA cycle and slower proliferation. In oleaginous yeasts, nitrogen limitation leads to the accumulation of metabolites directly upstream of α-ketoglutarate in the TCA cycle, such as citrate (Evans et al., 1983; Pomraning et al., 2016). Thus, available carbon skeletons together with rising pO_2_ and NADH levels appear to be crucial events that promote the onset of ASX synthesis. Like other oleaginous yeasts, *P. rhodozyma* possesses a citrate-malate shuttle for the translocation of citrate from the mitochondrial matrix to the cytosol (Fig. 2). Citrate is considered the most important source of acetyl-CoA for fatty acid synthesis in oleaginous yeasts grown that are grown under nitrogen deficiency (Davoli et al., 2004; Ratledge & Wynn, 2000). The cleavage of cytosolic citrate is catalyzed by ATP:citrate lyase (ACL), across different kingdoms, yielding cytosolic acetyl-CoA and oxaloacetate. ACL activity forms an important gateway between the glycolysis/TCA cycle and anabolic pathways because acetyl-CoA molecules are the building blocks for fatty acid synthesis (Bauer et al., 2005; Verschueren et al., 2019). This enzyme is found in several yeast species, and is consistently found in oleaginous yeast (Ratledge & Wynn, 2000, 2002). In batch cultures of *P. rhodozyma*, ACL activity has been shown to be notably upregulated, in response to the increase in pO_2_ in the culture medium that occurs upon the exhaustion of nitrogen. The accumulation of carotenoids and lipids also occurs upon nitrogen exhaustion and is paralleled by an increase in ACL activity, suggesting that this activity supplies acetyl-CoA for both, fatty acid, and carotenoid synthesis (Chávez-Cabrera et al., 2010; Martinez-Moya et al., 2011). *Rhodotorula gracilis* mutants that are deficient in ACL, which include carotenogenic yeasts that are phylogenetically related to *P. rhodozyma*, are also deficient in lipids and carotenoids (Shashi et al., 1990; Venkateswaran et al., 1992). The regulatory mechanism in *P. rhodozyma* has not yet been established. However, ACL activity from *Yarrowia lipolytica* has been reported to be upregulated by phosphorylation during nitrogen limitation (Pomraning et al., 2016).

The molecular form of available nitrogen in yeast cultures is a prominent determinant of cell growth rate, which can vary significantly depending on the source of nitrogen (Airoldi et al., 2016; Godard et al., 2007). For many cell types, growth in poor nitrogen sources impacts similarly the distribution carbon flow and leads to the accumulation of neutral lipids that are stored as lipid droplets (Aguilar et al., 2017). Some nitrogen compounds that support slow growth can compel yeast cells to release nonmetabolizable carbon compounds such as fusel oils (Godard et al., 2007).

The study of different nitrogen sources for ASX production has often led to the use of slowly metabolized amino acids such as valine, methionine, isoleucine, and phenylalanine, which usually diminish both the specific growth rate and the biomass yield on sugar (*Y*_X/S_) in relation to those under the use of ammonium salts (Meyer & du Preez, 1994b; Meyer et al., 1993; Wang et al., 2019). It seems reasonable to hypothesize that the use nitrogen sources that are metabolized slowly, as argued for ammonium limitation, may result in; low rate of protein synthesis, a low ATP usage (or low ATP demand), and an ADP deficit leading to impaired oxidative phosphorylation. Impaired oxidative phosphorylation theoretically results in impaired pmf usage for ATP synthesis and the consequent build-up of the pmf which by a feedback mechanism negatively affects the electron flow through the mETC. The increase in pO_2_ and the NADH/NAD^+^ ratio may encourage ROS production and thus promote ASX synthesis in *P. rhodozyma* cells grown in poor nitrogen sources. This agrees with the experimental data showing that the use of valine as the sole nitrogen source increases ASX production, but severely decreases the maximum specific growth rate and cell yield coefficient, *Y*_X/S_ (Meyer et al., 1993). This interpretation is also consistent with other published data (An et al., 1989; Barbachano-Torres et al., 2014; Johnson & Schroeder, 1996). For instance, the ASX hyper-producing mutants isolated by An et al. (1989) grew slower on ammonia, glutamate, or glutamine as nitrogen sources compared with the parental strain and also had lower *Y*_X/S_ when grown on several carbon sources. In addition, Xiao et al. (2015) found an inverse relationship between cellular ASX content and protein synthesis, in addition to competition effects between fatty acid synthesis and ASX synthesis in several ASX-overproducing mutant strains of *P. rhodozyma.* Intriguingly, Miao et al. (2021) have recently shown decreased growth, as well as reduced syntheses of fatty acids, DNA and RNA of the mutant strain MK19 when grown at high temperature (28°C vs. 25°C). However, ASX and ergosterol both were increased, ∼2-fold higher than levels at 21 or 25°C.

### Phosphate Limitation

Inorganic phosphate is an essential nutrient that is required for the biosynthesis of adenine nucleotides (including ATP, ADP, and AMP), phospholipids and metabolites used in energy metabolism (Yadav et al., 2016). Previous studies have shown that the end concentrations of biomass, ASX and total carotenoids, in *P. rhodozyma* cultures exhibit a similar bell-shaped relationship with the initial concentration of phosphate (0.3–9.7 mM) in the medium (Flores-Cotera et al., 2001). The maximum concentrations of biomass, ASX and total carotenoid pigments (11.1 mg/ml, 2.4 μg/ml and 4.5 μg/ml, respectively) were observed at approximately 0.65–1.3 mM phosphate. In contrast, the ASX content in yeast cells and the ASX/total pigment ratio both decreased progressively as the initial phosphate concentration increased (0.3–9.7 mM). The ASX content in yeast cells and ASX/total pigment ratio were highest at 0.3 mM phosphate, which was the lowest phosphate level tested. However, this phosphate level negatively affected growth and showed the lowest cellular protein content, but relatively high cellular fatty acid content (Flores-Cotera et al., 2001). It is striking that this challenging condition, which restricts both yeast growth and protein synthesis, still promotes the cellular accrual of ASX, which highlights the superlative priority given to cellular ASX accrual as an antioxidant defense under these conditions. The diminished cellular protein suggests that impaired protein synthesis may be crucial to trigger ASX synthesis in *P. rhodozyma* cells that are grown under low initial phosphate concentrations. Moreover, both impairments in the synthesis of proteins and nucleic acids, and diminished growth, have been reported as common outcomes when growing yeasts in phosphate-deficient media (Boer et al., 2010; Callieri et al., 1984).

Test performed on preliminary batch cultures in our laboratory indicated that phosphate deficiency (0.6 mM) in the medium adversely affects respiration of *P. rhodozyma* NRRL-Y-10922 yeast cells as compared to those grown in sufficient phosphate (2.7 mM). The culture with limited phosphate, showed a minimum pO_2_ at 48% saturation with air (24 hr), whereas the culture with abundant phosphate displayed a minimum pO_2_ at only 8% saturation with air (20 hr) (Martínez–Sanchez, 2018). This suggests that shortly after a phosphate deficiency arises, the lack of phosphate primarily affects the phosphorylation reaction ADP + Pi to generate ATP, with other reactions that use phosphate as a substrate less affected (e.g., substrate-level phosphorylation in glycolysis, in which oxidation of a substrate molecule is directly coupled to ATP synthesis). In this case, when the ATP production is restricted by phosphate deficiency, the sequence of events that occurs can be theoretically rationalized as follows: (i) The diminished rate of ADP phosphorylation due to Pi deficiency leads to the impaired synthesis of ATP. (ii) The pmf increases due to the restricted use of pmf in ATP synthesis. (iii) The pmf controls by a feedback mechanism the electron flow through the mETC. (iv) The impaired electron flow leads to the impaired reduction of O_2_ to water in the terminal oxidation step of the mETC, resulting in higher pO_2_ levels. (v) Similarly, such an impairment would also lead to a decrease in the reoxidation of NADH to NAD^+^ and thus to a higher NADH/NAD^+^ ratio (Bakker et al., 2001; Boer et al., 2010; Janssen et al., 2002). High pO_2_ levels together with a high NADH/NAD^+^ ratio, as mentioned above, predispose the production of ROS and ASX, which is in accordance with the available experimental data.

The slower growth cited together with the lower intracellular protein of *P. rhodozyma* grown under limited phosphate most likely arises from an inadequate supply of energy for the synthesis of protein and/or DNA/RNA (Flores-Cotera et al., 2001). The impaired protein synthesis, together with impairments to the generation of new functional biomass, renders carbon skeletons available for the biosynthesis of fatty acids, carotenoids and sterols. All latter processes are nearly coincident with the onset of ASX accrual, regardless of whether *P. rhodozyma* cells grow under nitrogen- or phosphate-limiting conditions. In brief, impaired oxidative phosphorylation, regardless of whether it arises from ammonium or phosphate deficiency, slows down NADH reoxidation, thereby leading to a higher NADH/NAD^+^ ratio and increased pO_2_ levels, stimulation of the ROS production, and induction of ASX synthesis. To our knowledge, this interpretation is consistent with the data that is available to date.

It is interesting that this mechanism also appears to be operative in other cell types. For example, ATPase inhibitory factor 1 (IF1) is a physiological inhibitor of mitochondrial F_0_F_1_-ATP synthase in mammalian cells. Interestingly, IF1-mediated inhibition of F_0_F_1_-ATP synthase promotes the production of ROS, which switches on the expression of nuclear genes that facilitate adaptation to a restrained oxidative phosphorylation (García Aguilar & Cuezva, 2018). It is also notable that IF1 overexpression can reprogram the energy metabolism to enhance glycolysis by limiting the production of ATP via mitochondrial F_0_F_1_-ATP synthase. Downregulated expression of mitochondrial F_0_F_1_-ATP synthase is usually found in human carcinomas as compared to its expression in normal tissues (Esparza-Molto & Cuezva, 2018).

It seems most likely that primary signals implied in the induction of ASX and fatty acid accrual, as well as the induction of other adaptive responses in yeast cells that are deprived specifically from nitrogen and/or phosphate may be jointly connected with mitochondria ROS release and levels of adenine nucleotides such as AMP, ADP ATP, and amino acids. In *S. cerevisiae*, ROS production induces the expression of various defense genes that are involved in the oxidative stress response. The expression of many of these genes is coordinated by two transcription factors, Yap1p and Skn7p (Brombacher et al. 2006). In addition, the well-conserved SNF1 yeast protein kinase (AMPK in mammalian cells) may be implied through the modulation of gene expression that result from changes in the levels of AMP, ADP, and ATP (Hardie, 2014, 2018; Ross et al., 2016), whereas amino acids may participate through modulation of mTORC1. Such processes may have been among the earliest signaling pathways to have arisen during eukaryotic evolution and all are highly conserved.

### Magnesium Limitation

Little data is available concerning the production of ASX under conditions in which magnesium is limited. However, some interesting insights can be gained from these data when examined together with information on the effect that limiting Mg has on other types of cell. Preliminary batch cultures of *P. rhodozyma* NRRL-Y-10922 were performed using a synthetic medium with the addition of either MgSO4·7H_2_O at 0.6 mM (+Mg) or 0.07 mM (−Mg) (Flores-Manzanero, 2018). Limiting the amount of available Mg increased the maximum cellular carotenoid content while decreasing the time taken to achieve this maximum (−Mg 0.62 μg/mg, 36 hr vs. +Mg 0.53 μg/mg, 90 hr). Nonetheless, this was at the expense of a decrease in (i) the maximum biomass concentration (−Mg 6.9 mg/ml vs. +Mg 9.5 mg/ml), (ii) the maximum protein concentration (3.3 mg/ml vs. 6.2 mg/ml), (iii) the total carotenoid concentration (3 μg/ml at 36 hr, vs 4.1 μg/ml at 66 hr), and (iv) the sugar consumption rate. In addition, the limited Mg adversely affected respiration in the *P. rhodozyma* cells; the culture with limited Mg showed a minimum pO_2_ level of 49% saturation in air after 24 hr, whereas the culture with plenty of Mg displayed minimum pO_2_ levels that were close to zero after 40 hr. These results are similar to those found in other studies in which yeast was grown in media with limited amounts of nitrogen, phosphate, or copper (Chávez-Cabrera et al., 2010; Flores-Cotera et al., 2001; Martínez-Cárdenas et al., 2018; Meyer et al., 1993). Nutrient limitations are therefore considered to adversely affect the maximum biomass, growth rate, oxygen consumption, protein concentration, and sugar consumption in cells.

Magnesium is an essential metal required for all biochemical reactions involving ATP. Limited Mg negatively affects the rate of these reactions (Pilchova et al., 2017). ATP and ADP bind with Mg to form Mg-ATP, and Mg-ADP, respectively, rendering them biochemically active (Pilchova et al., 2017; Touyz, 2004; Walker, 1994). Free Mg concentration is linked to the ATP/ADP ratio and free Mg builds up at low ATP/ADP ratios. Furthermore, Mg is necessary for glycolysis, the TCA cycle, F_0_F_1_-ATP synthase, protein and nucleic acid syntheses, ion transport and structural functions (Wolf & Cittadini, 2003). Mg stabilizes the secondary and tertiary structures of DNA and promotes DNA replication and transcription, and Mg deficiency may reduce DNA stability, protein synthesis, and mitochondrial function (Rowe, 2012). Low Mg concentrations adversely affect the catalytic activities of the F_0_F_1_-ATP synthase and the α-alpha-ketoglutarate dehydrogenase complex (cytosolic and mitochondrial forms) (Rodríguez-Zavala & Moreno-Sánchez, 1998). As a result, limited Mg can affect both oxidative phosphorylation and the functioning of the TCA cycle (Rodríguez-Zavala & Moreno-Sánchez, 1998). As cited above, deficiency in the function of the F_0_F_1_-ATP synthase may promote the production of ROS. Several studies have reported that dysfunctional α-ketoglutarate dehydrogenase is also associated with increased levels of ROS generation, which is linked to the redox imbalance that results from this condition (McLain et al., 2011; Tretter & Adam-Vizi, 2005).

Limiting the amount of available Mg also appears to cause the intracellular accumulation of greater amount of carotenoids. *P. rhodozyma* cells synthesize carotenoids in challenging oxidative environments (Chávez-Cabrera et al., 2010; Martínez-Cárdenas et al., 2018). This suggests that Mg deficiency could be associated with the increased generation of ROS. Shortly after Mg deficiency occurs, an insufficiently active Mg-ADP substrate may restrict oxidative phosphorylation. Alternatively, the small amount of Mg-ATP substrate available may result in insufficient ATP turnover and ultimately lower ATP synthesis, similar to that observed under nitrogen deficiency. This would be expected to lead to deficient oxidative phosphorylation in both cases, which then may lead to the build-up of pmf as is scarcely used in the synthesis of ATP, followed by pmf feedback control over the electron flow through the mETC, increasing both the NADH/NAD^+^ ratio and the pO_2_, stimulating ROS production and inducing the synthesis of ASX. This appears to concur with the scarce experimental data on *P. rhodozyma* cells that are grown under Mg-limited conditions.

Many studies with diverse cell types have found that an insufficient supply of Mg diminishes the rates at which biomass forms (Hauer-Jákli & Tränkner, 2019; Ikari et al., 2011). Other studies have shown that Mg deficiency can cause dysfunction in the key steps of oxidative phosphorylation and the TCA cycle thus encouraging higher ROS generation and oxidative stress (Ferre et al., 2010; Kramer et al., 1994; Kolisek et al., 2015; McLain et al., 2011; Shigematsu et al., 2018; Tretter & Adam-Vizi, 2005; Yang et al., 2006; Zheltova et al., 2016). Interestingly, Mg deficiency has been reported to decrease total protein concentration and repress amino acid biosynthesis in plant cells (Li et al., 2017; Peng et al., 2015). Weanling rats that were fed with Mg-deficient diets showed an elevated proportion of triacylglycerols whereas proteins and cholesterol were both reduced (Gueux et al., 1995). Limited Mg has also been linked with decreased cell viability (Martin et al., 2003; Yang et al., 2006) and the activation of uncoupling protein-mediated redox signaling, which decreases superoxide formation through pmf dissipation (Ježek et al., 2018). All these results appear analogous regardless of the cell type. Therefore, it may be true that the mechanism leading to all these responses in different cell types might be analogous to the above proposed mechanism by which *P. rhodozyma* adaptively responds to Mg deficiency.

### Copper and Iron Limitation

The three preceding sections indicate that dysfunctional oxidative phosphorylation that results from deficiencies in N, P or Mg, or other means, promotes the induction of ASX synthesis in *P. rhodozyma*. It is well known that ASX accrual can also be induced by impairing the electron flow through the cytochrome pathway of the mETC (An et al., 1989; An & Johnson, 1990; Johnson et al., 1994; Martínez-Cárdenas et al., 2018; Schroeder & Johnson, 1995a,b). The following four sections further detail different approaches in which ASX synthesis can be triggered by impairing the electron flow through the cytochrome pathway of respiration.

Martínez-Cárdenas et al. (2018) carried out batch cultures of *P. rhodozyma* NRRL-Y-10922 in a chemically defined medium with 7 μM Cu^2^^+^ (abundant copper), or 0.12 μM Cu^2^^+^ (limited copper). Limiting the amount of Cu available had a set of favorable effects on (i) the ASX content of the yeast cells, (ii) the ASX concentration in the culture broth, (iii) the final concentration of carotenoids, (iv) the proportion of ASX/total carotenoids produced, and (v) alternative oxidase expression (*aox*). Copper deficiency was also found to promote alcoholic fermentation under aerobic conditions in the wild-type strain of *P. rhodozyma*, with ethanol reassimilation observed later in the experiment despite the presence of sugars in the culture broth. Moreover as discussed above, nutrient deficiency in *P. rhodozyma* leads primarily to a decrease in the growth rate and the end biomass concentration. These effects were also apparent under copper deficiency; in fact, the growth rate, biomass concentration, and biomass yield on sugar (*Y*_X/S_) showed an inverse relationship with the ASX content in the cells (Flores-Cotera & Sánchez, 2001). In addition, limited copper adversely affected (i) the oxygen uptake rate, (ii) the specific sugar uptake rate, and (iii) the rate at which ethanol was reassimilated (Martínez-Cárdenas et al., 2018). Lower oxygen uptake rates entail higher average pO_2_ in yeast cultures and presumably more oxidative milieu inside cells. This was evidenced by the fact that the pO_2_ never dropped below a minimum of 23.4% (39 hr) of saturation with air throughout the culture with limited copper, whereas the culture with abundant copper displayed minimum pO_2_ levels close to zero (27–31 hr). Moreover, an unexpected pronounced *aox-*expression occurred both in the early growth period (12–18 hr), when sugars and pO_2_ were at high levels and later while cells were reassimilating ethanol (48–54 hr). These effects were observed under both copper deficiency and sufficiency, but the effects were remarkable under copper deficit. Notably pigmentation appeared much earlier in the yeast cells that were cultured under low copper concentration than in those grown under high copper concentration, indicating that carotenoid synthesis is associated with growth under copper deficiency.

Copper is an essential micronutrient that is toxic when in excess. Copper is typically associated with two mitochondrial cuproenzymes, superoxide dismutase (Cu/Zn-SOD, also Sod1) and cytochrome *c* oxidase (COX, complex IV) and the copper-binding COX assembly proteins Cox11, Cox17, and Sco1 (Cobine et al., 2004). COX, which is the terminal oxidase of the mitochondrial respiratory chain, accepts electrons from cytochrome *c*, to ultimately reduce oxygen to water, supporting the proton gradient that is required to generate ATP (Casteilla et al., 2001). COX has two unique catalytic bimetallic heme-copper sites (CuA and CuB), which are notably similar in all cell types (Babcock & Wikstrom, 1992; Horn & Barrientos, 2008; Popovic et al., 2010). Copper deficiency negatively affects the content and activity of COX in yeast cells, thereby impairing the electron flow through the mETC and oxygen consumption (Downie & Garland, 1973; Flores et al., 2000; Light, 1972; Nittis et al., 2001; Rossi et al., 1998). Accordingly, cellular growth is negatively affected by copper deficiency because of insufficient ATP production. In addition, the impaired electron flow that results from copper deficiency curtails the reduction of oxygen to water. Lower oxygen consumption leads to higher average pO_2_ levels in the culture, and more severe oxidative conditions for the cells. Moreover, since NADH oxidation mostly occurs through the mETC under aerobic conditions, a copper deficit inevitably slows down NADH reoxidation and increases the intracellular NADH/NAD^+^ ratio, as observed in human cells that lack COX (Sung et al., 2010). It is significant that wild-type *P. rhodozyma* cells initiate the intracellular accumulation of ASX shortly after copper limitation occurs. Also, it is remarkable that this yeast can accumulate ASX conditionally in a growth-associated fashion, that is, under copper deficiency, whereas yeast that is grown in a balanced medium generally accrues ASX in the late log growth phase and the early stationary growth phase in a non-growth associated fashion.

Copper deficiency promotes aerobic fermentation in *P. rhodozyma* cells (Martínez-Cárdenas et al., 2018). In addition, it is well established that hypoxic conditions promote the switch from respiration to fermentation in different cell types. Nevertheless, hypoxic conditions as well as copper deficiency both impair the rate at which the reduction of oxygen to water occurs, as well as the electron flow through the mETC. In the former instance, the oxygen reduction is affected because of the insufficiency of oxygen as a substrate/electron acceptor. In the second instance, the oxygen reduction rate is affected because either the catalytic activity or the amount of COX is insufficient. Hypoxia as well as copper deficiency impairs the electron flow through the mETC and likely lead to redox imbalances. Increasing NADH/NAD^+^ ratio has been reported *in vivo* for different cell types that are exposed to graded hypoxia (Mayevsky & Chance, 1982). A growing body of research has revealed a transient increase in the generation of ROS generation (by the mETC) during hypoxia, and these ROS appear to regulate the activation of protective mechanisms in diverse cell types, including an increase in the utilization of glucose (Chandel et al., 1998, 2000; Guzy et al. 2005; Hamanaka et al., 2016; Kwast et al., 1999; Poyton et al., 2009). Thus, we assume that a sudden increase in the NADH/NAD^+^ ratio occurs shortly after hypoxia arises. More reduced electron carriers must result in increased O_2_^•–^/H_2_O_2_ generation, despite the low pO_2_, which might serve to signal the existence of hypoxic conditions. The small increases in the expression of alternative oxidase (see next section on AOX) and astaxanthin synthase (encoded by *asy*, formerly *crtS*) detected by Martínez-Cárdenas et al. (2018) from 30 to 36 hr in cultures with abundant copper are also in agreement with the above interpretation. It is well accepted that alcoholic fermentation promotes NADH reoxidation, whereas the deactivation of fermentation reduces NADH turnover. Accordingly, aerobic and anaerobic fermentation are activated as a reflection of the increased cellular demand for NAD^+^ to cope the ATP turnover (Luengo et al., 2021).

It is intriguing that alcoholic fermentation, regardless of whether aerobic (−Cu) or anaerobic (hypoxia), as well as the expression of *aox*, and ASX synthesis, can all be triggered by redox imbalances, albeit under rather distinctive circumstances. Alcoholic fermentation, mitochondrial respiration, and AOX function all represent crucial components that act together in preserving the overall redox homeostasis and prevent severe oxidative stress in the yeast cells. Either aerobic or anaerobic fermentation promote cytosolic NADH reoxidation and glucose consumption, whereas AOX activity encourages mitochondrial NADH reoxidation.

According to our current analysis, it is striking that intracellular ASX accrual can be prompted by the impairment of any of the two branches of mitochondrial respiration. The impairment of oxidative phosphorylation (by retarding the reaction ADP + Pi to give ATP), appears to occur as argued under ammonium or phosphate deficiency. In another way, impairing of the electron flow through the cytochrome pathway, most likely occurs under copper deficiency, and is therefore predicted to occur in mutants of this yeast with assembly defects that are associated with COX (Dominiak et al., 2018). As argued above, high pO_2_ levels together with impaired NADH reoxidation are predisposed and suitable for generating intracellular ROS in *P. rhodozyma*. Yeast mutants that lack the proteins Cmc1 and Coa4, which lead to assembly defects of COX have been reported to increase the production of H_2_O_2_, hampering cell proliferation (Bode et al., 2013). However, the growth of these yeast mutants is significantly improved by the addition of dithiothreitol or glutathione. Moreover, the partial inhibition of COX activity has been reported to stimulate mitochondrial H_2_O_2_ production in houseflies (Sohal, 1993).

Iron limitation also significantly reduces the concentration of cytochromes in yeast cells (Light & Garland, 1971). Therefore, it might be expected that low Fe^2+^ concentrations (<1 mM), as cofactors in cytochromes, catalases, flavoproteins, and ferredoxins, could stimulate an increase in the intracellular ASX accrual in *P. rhodozyma* (Flores-Cotera & Sánchez, 2001). However, this was not the case, as iron deficiency decreased both the growth and the ASX content of the cells. The ASX synthase of *P. rhodozyma* belongs to the cytochrome P450 3A subfamily (Ojima et al., 2006). *In silico asy* analysis revealed the characteristic heme-binding site. It is also known that the reactions involved in the conversion of β-carotene into ASX in photosynthetic bacteria are catalyzed by diiron hydroxylases and ketolases, which require O_2_ for their activity (Fraser et al., 1998; Martín et al., 2008). These data appear consistent with the differential behaviors observed in *P. rhodozyma* when grown in limited iron versus copper-limited conditions.

### Respiratory Inhibitors and Alternative Oxidase

*P. rhodozyma*, as other carotenogenic yeast species, including *Cryptococcus, Rhodotorula, Yarrowia* and *Sporobolomyces*, as well as many filamentous fungi, possess an alternative oxidase (AOX) is insensitive to common cytochrome inhibitors such as cyanide, antimycin A, myxothiazol, and sulfur-containing amino acids (Chae & Nargang, 2009; Goffeau & Crosby, 1978; Henry & Nyns, 1975; Johnson & Schroeder, 1996; Joseph-Horne et al., 2001; Veiga et al., 2003a,b; Shiraishi & Fujii, 1986). AOX provides an alternative pathway through which electrons can pass in yeasts that are grown under severe copper deficiency, permitting the simultaneous reduction of O_2_ to H_2_O and continued growth (Downie & Garland, 1973; Martínez-Cárdenas et al., 2018). Although limiting the availability of copper may inhibit the electron transport that occurs through the cytochrome pathway, it simultaneously encourages AOX activity in several other yeasts as well as in *P. rhodozyma*. Copper depletion has been linked to the switch from standard to AOX respiration in the fungus *Podospora anserine* and the yeast *Candida utilis* (Downie & Garland, 1973; Servos et al., 2012). Therefore, AOXs bypass respiratory complexes III and IV by directly conveying electrons from QH_2_ to O_2_ (Dogan et al., 2018).

Johnson et al. showed that antimycin A, which is widely used to augment ROS generation from the Qo site of complex III, can improve ASX production and increase the ASX/total carotenoids ratio in *P. rhodozyma* (An et al., 1989; An & Johnson, 1990; Johnson et al., 1994; Schroeder & Johnson, 1995a,b). Antimycin A inhibits the oxidation of QH_2_ when attached to the Qi site of complex III (the site to which QH_2_ normally binds) and interrupts the electron transport through cytochromes. Consequently, the stimulation of ASX synthesis can be accomplished by nonspecifically blocking the electron transport at either complex III (by antimycin A) or complex IV (by copper deficit or defects in the COX assembly). It is known that complex III produces significant amounts of O_2_^•–^ from the reaction of O_2_ with the ubisemiquinone that is bound to the Qo site. The O_2_^•–^ is released from complex III toward both sides of the inner membrane (Murphy, 2009, and references therein). The positive effect on ASX synthesis therefore appears to be a result of the intracellular oxidative stress elicited by antimycin A. Because the respiratory oxidation pathways of NADH and FADH_2_ in yeast converge at the coenzyme Q pool, highly reduced pools of these electron carriers (high NADH/NAD^+^ and QH_2_/Q ratios) may be expected to result from blocking the respiratory chain via the use of antimycin A, or by other means downstream of complex III, which has previously been shown to positively influence O_2_^•–^ generation via one-electron reduction (Dominiak et al., 2018; Herrero et al., 2008).

The results of these studies suggested that ASX synthesis occurs in the presence of antimycin A, along with a shift from cyanide-sensitive respiration to cyanide-insensitive respiration. Accordingly, AOX activation has been proposed to be closely associated with ASX synthesis; however, a later study indicated that deleting *aox* increases the cellular content of ASX, signifying that AOX is not essential for ASX synthesis (Hoshino et al., 2005). Apart from the above-mentioned studies, the AOX in *P. rhodozyma* has not been characterized in detail, but many AOXs have been characterized in other organisms such as yeasts, fungi, animals, and plants. The AOXs in plants show considerable homology with those in fungi (Guerrero-Castillo et al., 2012; Helmerhorst et al., 2005). Disruption of the mETC leads to the induction of AOX in several of these organisms. AOXs in plants have much lower affinity for O_2_ than COX; therefore, AOX activity may rapidly decrease under conditions of low pO_2_ levels and hypoxia (Rhoads et al., 2006).

**Fig. 3:**
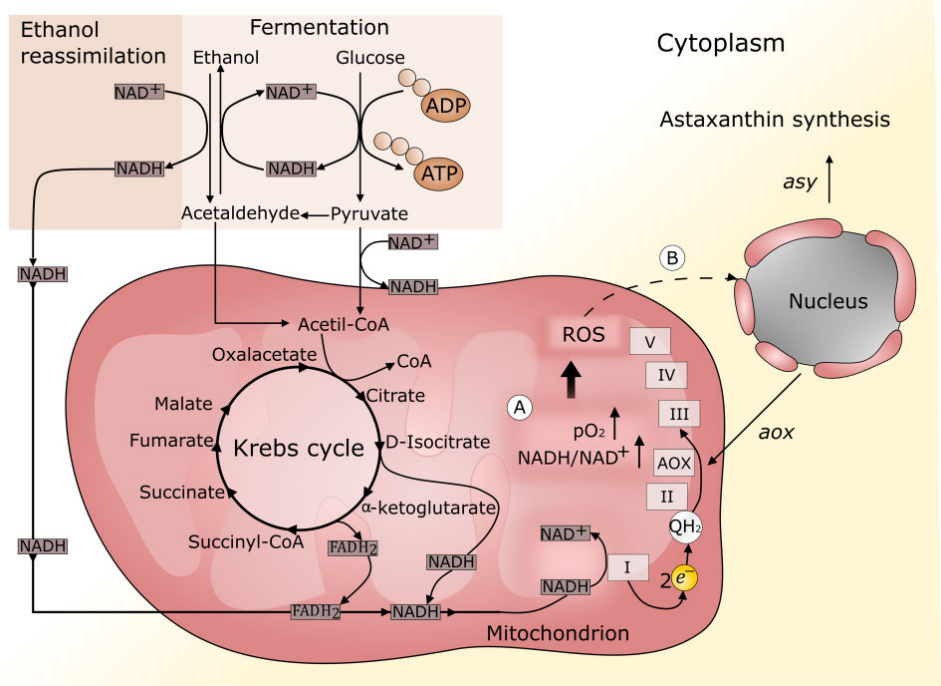
Primary reactions involving the NADH/NAD^+^ couple. Glycolysis, TCA cycle and ethanol reassimilation are key processes to provide NADH, whereas reoxidation of NADH mainly takes place at complex I in the respiratory chain or via the fermentation of pyruvate to ethanol. The ASX-accrual in *P. rhodozyma* cells arises under oxidative conditions if greater provision of NADH relative to NADH reoxidation occurs. For example (A) Impairment of the electron transport in the mETC elicits higher NADH/NAD^+^ ratio and high pO_2_ levels. These conditions predispose greater ROS generation, as a result. (B) Greater ROS production boosts ROS signaling that serves to activate an adaptive response including ASX synthesis as well as *asy* and *aox-*expression. Other conditions such as ethanol reassimilation can result in redox imbalances that promote ASX-accrual in cells. Alcoholic fermentation, mitochondrial respiration, and AOX function, all represent crucial components acting in concert to perform NADH oxidation, preserve overall redox homeostasis and prevent severe oxidative stress on the yeast cells. Both aerobic and anaerobic fermentation promote cytosolic NADH oxidation and glucose consumption, while AOX activity encourages mitochondrial NADH oxidation. In *S. cerevisiae*, increased heme synthesis has been shown to induce the metabolic switch from fermentation to respiration even under conditions of glucose repression (Zhang et al., 2017).

All AOXs have a binuclear iron center that catalyzes the oxidation of QH_2_ while reducing molecular O_2_ to H_2_O. Thus, QH_2_, the reduced form of Q, may donate its electrons to AOX or, alternatively, to COX via complex III, meaning that electrons from QH_2_ can be partitioned freely over both pathways (Fig. 3). AOX activity is also dependent on the QH_2_/Q ratio (Chae & Nargang, 2009; Hoefnagel and Wiskich, 1998; Van Aken et al., 2009; Yoshida et al., 2007). Moreover, inhibition of the electron flow through the mETC, regardless of whether by external agents or mutations, is known to increase ROS production due to the accumulation of reduced electron carriers such as NADH or QH_2_ (Demasi et al., 2006; Fang and Beattie, 2003; Guerin et al., 1989; Hoefnagel & Wiskich, 1998; Millenaar et al., 1998; Minagawa et al., 1992; Sakajo et al., 1993, 1997; Zhao et al., 1996). Accordingly, AOX is induced by ROS generators such as menadione and paraquat in *P. rhodozyma*, and is inhibited by free radical scavengers (An & Johnson, 1990; Johnson & Schroeder, 1996). Other known inhibitors of AOX include hydroxamic acids (e.g., salicylhydroxamic acid, SHAM), disulfiram, and *N*-propyl gallate without distinction of the organism (Akhter et al., 2003; Veiga et al., 2003a; Wagner & Moore, 1997). The biochemical role of AOX in yeast is not completely understood, but it has been proposed to function as an energy-overflow mechanism when the electron flow is hindered (Flores et al., 2000). AOX activity has been associated with other possible functions in different cell types, such as (i) dissipating the NADH surplus caused by rapid catabolism when the mETC is impaired or when the ATP demand is low, (ii) stabilizing the redox state of the mitochondrial enzyme components to permit continued activity, (iii) restricting the mitochondrial ROS production thereby reducing oxidative damage to cellular components, and (iv) allowing cell growth when the mETC is impaired, since it permits the translocation of protons from the mitochondrial matrix to the intermembrane space from complex I, and thus ATP synthesis (Akhter et al., 2003; Maxwell et al., 1999; Millenaar & Lambers, 2003; Moore & Albury, 2008; Guerrero-Castillo et al., 2012). In general, yeasts that possess an AOX always possess complex I in their mETC (Li et al., 2009, 2011; Veiga et al., 2003a,b). The electron transfer from NADH to molecular O_2_ via AOX, which itself does not pump protons; still permits proton translocation from the mitochondrial matrix to the intermembrane space, at complex I, thus AOX activation decreases the respiratory energy output (Maxwell et al., 1999; Veiga et al., 2003a). Nevertheless, the translocation of protons from a single respiratory complex still permits metabolism to continue when the mETC is disrupted (de Vries & Marres, 1987). In *P. rhodozyma*, the progressive increase in ASX synthesis under diminishing copper concentrations, with the associated decrease in the cellular biomass and *Y*_X/S_, suggests a gradual impairment of the mETC (Flores-Cotera & Sánchez, 2001). Since cellular biomass and *Y*_X/S_ under oxidative conditions are largely determined by the number of proton translocation sites, these results indicate that cells obtain less energy from the carbon source when copper is limited, suggesting a gradual activation of ASX synthesis and AOX when the mETC, which is energetically most favorable, is disrupted (Verduyn, 1991). In other words, ASX synthesis and AOX are suitably activated by a common driving force, namely a NADH surplus (Martínez-Cárdenas et al., 2018). Uncoupling proteins (UCPs) may assume a function similar to that of AOX in other cell types. UCPs have QH_2_ as an obligatory companion, and their function serves to modulate mitochondrial ROS production (Casteilla et al., 2001).

More recently, Pan et al. (2020) showed that the phyohormone 6-benzylaminopurine (6-BAP) impairs the TCA cycle function and encourages ASX and fatty acid syntheses. ROS analysis suggested that 6-BAP increases ROS generation, accounting for the improved ASX production observed.

### Mutant Strains

Several research groups have now isolated mutant strains of *P. rhodozyma* with higher cellular ASX content than their parental strains. Mutant strains have been obtained using physical and chemical agents such as UV light, Co60, ozone, ethylmethanesulfonate, 1-methyl-3-nitro-1-nitrosoguanidine, hydrogen peroxide, hypochlorous acid, and antimycin A (An et al., 1989; An & Johnson, 1990; Calo et al., 1995; Fang & Chiou, 1996; Gong et al. 2012; Kim et al., 2004; Schroeder & Johnson, 1995b). The ASX content in wild strains has been shown to be between 200 and 300 μg/g (total carotenoids/yeast biomass). In contrast, some mutants can accumulate up to 3000 μg/g, yet the measurement of individual cells indicates that levels up to 15,000 μg/g can be achieved (Johnson & An, 1991). Nearly all these ASX-hyperproducing mutant strains show impaired respiration, slower growth, form smaller colonies, and show lower yields (*Y*_X/S_) in different carbon sources as compared to their respective parental strains (Lodato et al., 2007; Ukibe et al., 2008). In addition, high producers are often unstable, and the precise nature of the mutations has generally not yet been elucidated. Nevertheless, the data obtained indicate that the mutant strains harness less energy from the carbon source. (An et al., 1989; Johnson & An, 1991; Lodato et al., 2007; Meyer et al., 1993; Miao et al., 2010, 2019; Visser et al., 2003). In contrast to native strains, which mostly synthesize ASX in a non-growth-associated manner, a notable characteristic of many mutant strains is their ability to synthesize ASX in a growth-associated mode. ASX synthesis appears to be deregulated even in the early stages of growth in batch cultures of mutant strains when relatively high sugar levels prevail (Fang & Cheng, 1993; Fang & Chiou, 1996; Johnson & Lewis, 1979; Meyer & du Preez, 1994a,b; Miao et al., 2019; Ukibe et al., 2008). For instance, Miao et al. (2010) utilized 1-methyl-3-nitro-1-nitrosoguanidine and Co60 mutagenesis to isolate a slow-growth mutant strain of *P. rhodozyma* (strain MK19) that is capable of accumulating 17-fold more ASX than the parental strain. ASX synthesis was found to be associated with growth when the mutant strain was cultured with glucose as carbon source, in contrast to the parental strain that produced ASX at the end of the log growth phase. The genes involved in ASX synthesis in the mutant strain were overexpressed and detectable from the earliest phases of growth, with *crtE* increasing 2- to 7-fold; and *crtI, crtYB*, and *asy*, from 7- to 29-fold as compared to the wild type, depending on the time of analysis. Moreover, Lodato et al. (2007) reported that the transcripts of *crtE, crtYB, crtI*, and *asy* in another mutant strain were approximately 2-fold greater in the early log growth phase than the parental strain. Conversely, several wild-type strains quickly accumulate ASX at the end of the log-growth phase, particularly after sugars become depleted from the culture medium (Alcaíno et al., 2016; Chávez-Cabrera et al., 2010; Cordova et al., 2016; Hu et al., 2005,2007; Johnson & Schroeder, 1996; Kusdiyantini et al., 1998; Liu & Wu, 2007; Miao et al., 2010; Parajó et al., 1998; Schmidt et al., 2011; Zheng et al., 2006).

It can be expected that mutant strains with distinctive mutations affecting genes that encode for components of the respiratory chain (either in oxidative phosphorylation or downstream complex III in the cytochrome pathway), TCA cycle, or nitrogen assimilation largely adopt a growth-associated ASX synthesis mode. Furthermore, any of these mutations can lead to an impairment of both the reoxidation of NADH and the reduction of oxygen to water. This could predictably increase the NADH/NAD^+^ ratio and pO_2_ levels promoting ASX synthesis from the early growth phase of *P. rhodozyma*. We hypothesize that numerous distinct metabolic impairments that inhibit the cell growth in mutant strains can encourage ASX synthesis from the early stages of growth. For instance, mutations in the mitochondrial MTATP6 gene that encode subunit 6 of the F_0_F_1_-ATP synthase of the budding yeast might be suitable for improving ASX synthesis (Niedzwiecka et al., 2018). Two mutations in the ATP6 gene that are related to human cancer affect ROS, calcium homeostasis and the mitochondrial permeability transition in yeast. Mutations and deletions in mtDNA can promote cellular oxidative stress, mitochondrial dysfunction, and cell death (Roubicek & de Souza-Pinto, 2017). Thus, mtDNA may be a potentially relevant target for improving ASX production. Several reports have suggested that the mutations that are involved in nitrogen assimilation or others that impair the mETC are accountable for ASX overproduction in some mutant strains (An et al., 1989; Barbachano Torres et al., 2014). However, the fact that the overproduction of ASX may still require the suppression of feedback regulation by an intermediary or end product over the carotenoid synthetic pathway and avoidance of sugar repression requires consideration (Alcaíno et al. 2016; Cordova et al., 2016; Johnson & Schroeder, 1995a, 1995b; Miao et al., 2019).

### Photoregulation of Carotenogenesis

Carotenoids perform several important physiological functions in all living organisms. In nonphotosynthetic organisms, carotenoids protect cells against the ROS that are generated by photo-oxidative processes and normal respiration. Protection against ROS is performed by quenching singlet oxygen (a nonradical ROS), the excited states of photosensitizing molecules, and the scavenging of free radicals (Domonkos et al., 2013).

Carotenogenesis is regulated by light in numerous fungi and other cell types. Several photo-inducible enzymes are known to be carotenogenic enzymes, including HMG-CoA reductase and ASX synthase (An & Johnson, 1990; Bhosale, 2004; Sandmann, 1994). These enzymes are largely induced by blue light; however, the regulatory mechanism is poorly understood in *P. rhodozyma* (Visser et al., 2003). Light influences both the cell growth and the intracellular carotenoid content of *P. rhodozyma* cells. High-intensity light inhibits growth and decreases the cellular carotenoid content of the yeast cells on agar plates, probably because continuous light exposure overwhelms the antioxidant defenses of the cells (An & Johnson, 1990). It is well known that individual cells can tolerate strong light incidence when exposed intermittently, as occurs in shake flasks or bioreactor suspended cultures. In these cultures, each individual cell is exposed to dark/light cycles rather than uninterrupted exposure as a result of random liquid motion. Therefore, light may cause stress and impair growth, but has a stimulating effect on ASX synthesis in comparison to cultures that are grown under dark conditions (An & Johnson, 1990; Bhosale, 2004; Breitenbach et al., 2011; de la Fuente et al., 2010; Frengova & Beshkova, 2009; Meyer & du Preez, 1994c; Schmidt et al., 2011; Stachowiak, 2013).

Vázquez (2001) examined the effect of light on six *P. rhodozyma* strains grown in shake flasks with xylose as the carbon source. For each strain, the end carotenoid concentration was comparatively higher in the light than in the dark. In addition, each strain under light conditions produced greater ASX/total carotenoid ratio. However, the biomass concentration and *Y*_X/S_ were differently affected depending on the specific strain. The three strains with the lowest cellular content of ASX (110–207 μg/g) that were tested, under light showed lower cell biomass and *Y*_X/S_, presumably because the light-imposed stress overwhelms their low antioxidant capability. In contrast, the three strains with the highest content of ASX (224–242 μg/g) showed greater cell biomass and *Y*_X/S_, suggesting that they possess a more efficient photo protective capability to cope with light-stress. However, it should be considered that ASX accumulation in *P. rhodozyma* cells generally shows a bell shaped response to the intensity of ROS emission.

Certain molecules absorb light to form transient excited states. Singlet oxygen (^1^O_2_) is mostly generated by a photosensitized reaction, wherein a UV or visible light-absorbing molecule, the sensitizer, transfers the energy gained from excitation to molecular oxygen to generate the singlet activated form ^1^O_2_ (Clo et al., 2007; Davies, 2004; Khorobrykh et al., 2020). Photons, particularly those associated with blue light, interact with and excite cellular sensitizers such as iron–sulfur (Fe–S) metalloproteins. Many Fe–S clusters are known to be highly sensitive to oxidation. Oxygen-sensitive [Fe–S] cluster cofactors may act as sensitive sensors for oxygen or ROS (Outten, 2007). Under light-induced oxidative stress, some Fe–S clusters release Fe^2+^ and become disassembled, impairing both the electron flow through the mETC (assuming a slower rate of cluster synthesis) and respiration. This may lead to the accumulation of reduced electron carriers in the mETC (e.g., NADH, QH_2_) and increased pO_2_ levels. Then, the direct electron transfer from reduced forms of electron carriers to O_2_ generates both O_2_^•–^ and H_2_O_2_, further contributing to the oxidative process (Barros et al., 2004; Hamblin, 2018; Kim & Jung, 1992; Longo et al., 1999; Outten, 2007; Rigoulet et al., 2011; Robertson et al., 2013). In addition, Fe^2+^ catalyzes the formation of the highly reactive hydroxyl radical (OH•) in the presence of H_2_O_2_ and O_2_^•–^, which is perhaps the strongest oxidant known in biological systems. The malfunctioning of the iron–sulfur cluster assembly machinery in *S. cerevisiae* has been shown to induce oxidative stress via an iron-dependent mechanism, leading to dysfunction in the respiratory complexes (Gómez et al., 2014). Any of the above processes, may presumably encourage ASX synthesis in *P. rhodozyma* cells under light. Photo chemically generated ROS are known to induce the expression of numerous eukaryotic genes, including stress proteins (Ryter & Tyrrell, 1998). Another interesting possibility would be the use of Fe–S cluster-targeting drugs that induce ROS generation and the disassembly of Fe–S clusters, which may also promote ASX synthesis (Vernis et al., 2017).

In another study, two strains of *P. rhodozyma*, wild-type 67–385 and mutant ant-1–4, were grown in dark and light in culture medium containing 0.2 μM antimycin A (An & Johnson, 1990). When both strains were grown in darkness and in the presence of antimycin A, the maximum biomass declined by approximately one-third and one-half, respectively, relative to the control, which is presumably as a result of the specific inhibition of the mETC. Nevertheless, when these strains were exposed to light, this inhibition was removed; more biomass was produced by cells treated with light plus antimycin A than cultures that were treated with antimycin A in the darkness. In addition, in cells of the wild-type strain that were treated with antimycin A, light stimulated the accumulation of ASX (nearly 2-fold) in comparison to any of the cultures that were grown either under dark, light, or dark plus antimycin A conditions. The mutant strain grown in antimycin A plus light also showed increased ASX content in relation to cultures grown under light only. These data indicate that light allows yeast growth in cultures containing antimycin A while simultaneously stimulating ASX biosynthesis. It would be extremely interesting to know the mechanism by which light releases the inhibition of electron transfer through the mETC that is imposed by antimycin A. It is well established that photosynthetic systems can excite chlorophyll electrons when photons interact with the reaction center of the photosystem II. These light-energized electrons serve to reduce plastoquinone, the primary electron acceptor of light-driven reduction. Reduced plastoquinone passes the photo excited electrons to the b6–f complex, a proton pump embedded in the thylakoid membrane in chloroplasts that is able to pump protons into the thylakoid space to generate a proton gradient (pmf) across the membrane (Horak, 2020; Steinbeck et al., 2020). This reaction is analogous to the reaction catalyzed by cytochrome bc1 (complex III) in the mETC. The proton gradient (pmf) is also used in photosynthetic systems to drive the ADP photophosphorylation to form ATP by the F_0_F_1_-ATP synthase. Therefore, it is quite possible that some other mechanism, beyond that explained above (the light-induced disassembly of the Fe–S cluster and the release of Fe^2+^) might be involved in stimulating ASX synthesis and the growth of *P. rhodozyma* in the presence of antimycin A and light. It is yet unknown whether photo excited electrons could enter downstream of the blockage point imposed by antimycin A and drive proton pumping by COX. This means might explain the light driven release of inhibition of growth in antimicyn A treated cells. Given that carotenoids are pigments that absorb light in the spectral region in which the sun irradiates maximally, and that the associated energy is transferred to the chlorophylls, the previous statement may be a striking possibility. In photosynthetic systems, the limited capacity for the biochemical utilization of absorbed light energy could induce the formation of ROS such as O_2_^•–^ and H_2_O_2_ (Huner et al., 2012; Ruban et al., 2012). However, the reduction state of plastoquinone, in this case, could assume the central position of the NADH/NAD^+^ couple described throughout this work.

One mutant strain of *P. rhodozyma* (yan-1) was able to accumulate β-carotene, but not xanthophylls (An & Johnson, 1990). When it was cultured in medium containing antimycin A, the exposure to light released this deficiency, and the ability to produce xanthophylls was recovered; however, the mechanism involved remains unknown. In general, light inhibits the growth of mutant strains to a greater degree than wild-type strains. This suggests that a metabolic impediment in the mutants renders them more prone to generating ROS when exposed to light. The response to low light intensity appears to be similar in other microorganisms including fungi, algae, and bacteria, For example, *Rhodotorula glutinis*, a yeast that is phylogenetically close to *P. rhodozyma*, increases carotenoid production following exposure to weak white light, but at the expense of decreased growth (Bhosale, 2004; Tada, 1993).

### Fermentation and Ethanol Reassimilation

The preceding paragraphs describe different ways to induce the accrual of ASX in *P. rhodozyma* cells. Mechanisms that are dependent on restricting the reoxidation of NADH were primarily examined, namely, those that depend on the impairment of the electron flow through the mETC to promote the cellular accrual of ASX. In this section, we describe other different ways by which cell ASX accrual is induced, especially those that depend on the oversupply of reducing equivalents in the form of NADH to generate a redox imbalance. First, we briefly explain some of the fundamentals involved in the metabolism of ethanol. Second, we discuss the significant ASX accrual by *P. rhodozyma* cells that takes place as a result of an excessive supply of NADH reaching the respiratory chain and the greater NADH/NAD^+^ ratio that occurs under such conditions.

Glucose catabolism, which leads to the production of ethanol, is a common occurrence in *P. rhodozyma* that grows exponentially under high sugar concentrations and limited oxygen (Liu & Wu, 2007, Reynders et al., 1997; Yamane et al., 1997a). Low intracellular carotenoid content and little or no pigment accumulation occur under such respiro-fermentative conditions (Chávez-Cabrera et al., 2010; Johnson & Schroeder, 1996; Wozniak et al., 2011). The mRNA expression of genes that are involved in carotenogenesis are known to be relatively low in the presence of glucose (Marcoleta et al., 2011). However, when the fermentable sugar is exhausted, *P. rhodozyma*, like other facultative yeasts, turns to ethanol as a carbon source and the pO_2_ in the culture medium rises (Liu & Wu, 2008; Martínez-Cárdenas et al., 2018; Yamane et al., 1997b). Facultative yeast cells can switch very rapidly from anaerobic to aerobic growth once higher pO_2_ levels arise at the diauxic shift. Mitochondrial respiration is associated with widespread changes in the expression of the genes that are involved in fundamental cellular processes such as carbon metabolism, protein synthesis, mETC, and carbohydrate storage (DeRisi et al, 1997; Franco et al., 1984; Heinisch & Hollenberg, 1993; Konz et al., 1998; Slavov & Botstein, 2011). These activities are, to some extent coregulated, and induced upon the release of glucose repression (Alcaíno et al., 2016; de Vries & Marres, 1987; Marcoleta et al. 2011).

Ethanol metabolism requires active respiration; in fact the maximum rates of ethanol assimilation are dependent on the respiratory capacity of cells. Ethanol is oxidized via acetaldehyde/acetate/acetyl-CoA and supplies carbon skeletons to the TCA cycle in the form of acetyl-CoA (de Vries & Marres, 1987; Heinisch & Hollenberg, 1993). In addition, ethanol metabolism yields more NADH per carbon equivalent than glucose (Villadsen et al., 2011). Consequently, ethanol reassimilation in yeasts, and in many other cell types, increases the NADH/NAD^+^ ratio due to the excessive provision of NADH to the respiratory chain. In both yeasts or other types of cells, ethanol metabolism elicits cellular oxidative stress by increasing the formation of ROS (Jones, 1989; Hoek et al., 2002; Miñana et al., 2002; Sastre et al., 2007). The uncontrolled formation of ROS that may be linked to ethanol metabolism may promote the release of iron from Fe–S-containing enzymes, leading to apoptosis or cell death (Gomez et al., 2014; Pérez-Gallardo et al., 2013). Ethanol stress has been recently described as an activator of the UPR response in the yeast *Saccharomyces* (Navarro-Tapia et al., 2017). These facts suggest that ethanol metabolism causes substantial oxidative stress in cells from diverse origin. The depletion of NAD^+^ levels that is associated with ethanol reassimilation diminishes the activity of NAD^+^-dependent enzymes, including those associated with the TCA cycle (e.g., ICDH). Consequently, the TCA cycle function is compromised by NAD^+^ deficiency soon after ethanol assimilation begins (Hou et al., 2009; Jones, 1989; Luengo et al., 2021; Prieto et al., 1992; Slavov & Botstein, 2011; Watson et al., 2011). The compromised TCA cycle function then rapidly impairs the synthesis of amino acids, proteins, and nucleic acids, as well as respiration. Cell division is therefore delayed and growth slows down and may even cease once ethanol assimilation starts. A redox imbalance together with relatively high pO_2_ is expected to foster O_2_^•–^/H_2_O_2_ generation (Hoffman & Brookes, 2009; Miñana et al., 2002; Quinlan et al., 2012), leading to oxidative stress and the subsequent accrual of intracellular ASX. All these data seem to be in accordance with the greater accumulation of carotenoids that is generally associated with *P. rhodozyma* yeast cells grown with ethanol as a carbon source.

The onset of ASX synthesis in wild-type strains of *P. rhodozyma* frequently occurs shortly after the shift from glucose to ethanol as the major carbon and energy source. This typically occurs together with a transitory phase characterized by slower growth or even a decrease in the cellular biomass, the deceleration of protein synthesis, and a decrease in the intracellular protein content, as has been observed in other yeast at the diauxic shift (Chávez-Cabrera et al., 2010; Hosios & Vander Heiden, 2018; Johnson & Lewis, 1979; Jones, 1989; Lodato et al., 2007; Prieto et al., 1992). Considering that protein synthesis cannot be fully operative under a compromised TCA cycle, the provision of carbon skeletons for proliferation diminishes, thereby decreasing the energy demands. Accordingly, a surplus of carbon skeletons and ATP could be available for other biosynthetic processes distinct than those required for cell proliferation. We assume that, as a result of the elevated NADH/NAD^+^ ratio, the pmf could increase and exert feedback control over the electron flow through the mETC, alike as was recently reported for a somewhat equivalent situation observed in mammalian cells (Luengo et al., 2021).

Taken together, the events cited above highlight the significant stress conditions that are faced by cells when ethanol is used as carbon a source. Our previous study (Martinez-Cárdenas et al., 2018) showed that even small amounts of ethanol consumption, when coincident with impaired respiratory activity (e.g., due to conditions such as copper deficiency), entail conditions of substantial stress on *P. rhodozyma* cells. Ethanol reassimilation was found to encourage the intracellular accrual of ASX and pronounced *aox-*expression, regardless of the presence (3-fold) or deficiency (6.5-fold) of copper. These results are consistent with our inference that ethanol reassimilation results in a redox imbalance and severe oxidative stress in *P. rhodozyma* cells. Higher *aox-*expression is of vital importance, as it serves the imperative need to minimize the surplus of reducing equivalents, and thus limit the rate of intracellular O_2_^•–^/H_2_O_2_ generation (Maxwell et al., 1999; Vanlerberghea & McIntosh, 1997). The sharp drop in biomass (48–54 hr) observed (Martínez-Cárdenas et al., 2018) in a culture deficient in copper suggests significant cell death, which is consistent with the above interpretation. In contrast, yeast cells with abundant copper, with greater respiratory capacity, evade the drastic decrease in biomass (cell death) during the period of ethanol reassimilation (42–60 hr) by means of the intermittent expression of *aox* and the intracellular accrual of ASX. It is also of note that cells grown under copper deficiency have been found to yield a higher proportion of ASX/total carotenoids as compared to cultures with abundant copper (76% vs. 56%). This result reinforces the value of ASX as a unique gauge to assess the relative importance of distinct stressful conditions.

Ethanol reassimilation increases the abundance of carotenogenic proteins, redox- and stress proteins, and the intracellular accumulation of carotenoids and fatty acids (Martínez-Cárdenas, et al., 2018; Martinez-Moya et al., 2011). Lodato et al. (2007) reported maximum expression levels of *crtYB, crtI*, and *asy* genes associated with ethanol assimilation in a wild-type strain. A different report indicated that ethanol induces *crtYB* and *asy* gene expression and promotes the *de novo* synthesis of carotenoids (Marcoleta et al., 2011). Other researchers have reported enhanced carotenoid synthesis as a result of the exogenous addition of ethanol to *P. rhodozyma* cultures. Gu et al. (1997) reported increased levels of carotenoids when ethanol was added (0.2% v/v) at different growth stages. The activity of HMGR, a key enzyme in mevalonate synthesis, was stimulated by the addition of ethanol. Moreover, a sizeable increase in the intracellular ASX content (2–3-fold) or the ASX concentration in *P. rhodozyma* has been observed in cultures supplemented with ethanol or linked with ethanol metabolism (An et al., 1989; Kim et al., 2003; Kim & Chang, 2006; Liu & Wu, 2008; Yamane et al., 1997b; Zhu et al., 2001). ASX synthesis has also been stimulated in feed batch cultures that are fed with ethanol, although at the expense of slower growth compared to those using glucose as carbon source (Yamane et al., 1997b).

Chávez-Cabrera et al. (2010) found that increases in ACL activity, the key enzyme for the supply of acetyl-CoA into the cytosol, occurred jointly with ethanol assimilation, increasing pO_2_ in the culture broth and the intracellular accumulation of carotenoids. Accordingly, Cannizzaro et al. (2004) suggested that the supply of cytosolic acetyl-CoA for ASX synthesis in *P. rhodozyma* appears to originate from citrate rather than pyruvate.

### Other Conditions Leading to an Oversupply of NADH

Besides ethanol, it is interesting that the metabolism of other carbon sources, in other cell types, can lead to an excessive provision of reducing equivalents (either as NADH or QH_2_) to the respiratory chain and promote cellular oxidative stress. For example, high glucose levels may promote high carbon flow through the glycolytic and TCA pathways, providing excessive NADH reducing equivalents to the respiratory chain and resulting in cellular damage (Wu et al., 2016). Teodoro, Gomes, et al. (2013) showed that mitochondrial injury takes place early upon hyperglycemic insult and can lead to permanent, cumulative damage that might be one of the earliest causes of pre-diabetic conditions. Hyperglycemic-exposed cells show a high NADH/NAD^+^ ratio and high ATP levels with increased mitochondrial pmf and low ADP levels, and the mitochondrial respiratory chain components are heavily reduced. According to the authors, the damage is probably caused by an increase in the NADH/NAD^+^ ratio, which contributes to increased ROS production. Wu et al. (2016) suggested that the oxidative damage resulting from redox imbalances between NADH and NAD^+^ may be a major factor that contributes to the development of diabetes and its complications.

It is interesting that the early growth period (12–24 hr) of the *P. rhodozyma* cultures reported in our former publication (Martínez-Cárdenas et al., 2018), during which both sugar and pO_2_ were at high levels (25–30 g/l and 80–100%, respectively), were characterized by a fast specific sugar uptake rate (g sugar g cell-1 h-1), steady growth, a marked accumulation of cellular ASX and *aox*-expression. It is intriguing that ASX synthesis was active under the above conditions, despite the fact that glucose-dependent repression, which operates at the transcriptional level, is known to be functional in regulating carotenoid production in *P. rhodozyma* (Alcaíno et al., 2016; Córdova et al., 2016). A hypothetical explanation that fits with the above data involves a model in which a high specific sugar uptake results in an excessive supply of NADH that generates a redox imbalance, thereby leading sequentially to a higher NADH/NAD^+^ ratio, mitochondrial membrane hyperpolarization, feedback inhibition of the electron flow through the mETC, the consequential increase in pO_2_, and stimulation of ROS production, which is followed by *aox-*expression and ASX synthesis.

It is equally interesting that both anaerobic fermentation and aerobic fermentation are evidently activated by redox imbalances in *S. cerevisiae*. In the former instance, low pO_2_ levels restrict the electron flow through the mETC because of the slow reduction of molecular O_2_ to water in the terminal oxidation step of the respiratory chain (resulting from insufficient electron acceptor availability). Thus, the reoxidation of NADH is impaired, and the resulting redox imbalance can easily spread from the mitochondria to the cytoplasm to promote alcoholic fermentation (Gui et al., 2016; Noda et al., 2002; Rigoulet et al., 2004). Moreover, aerobic fermentation or mixed respirofermentative metabolism, which results in the unfavorable fermentation of glucose-derived pyruvate to ethanol despite ample oxygen availability, is associated with proliferation across many cell types and contexts (Koobs, 1972; Luengo et al., 2021; Martínez-Cárdenas et al., 2018). For instance, high glucose concentration in the culture medium may cause *S. cerevisiae* to switch from a purely oxidative to a mixed respirofermentative metabolism (Kasperski, 2008; Overkamp et al., 2000; Postma et al. 1989; Vemuri et al., 2007). As cited above, high glucose concentrations are linked with the excessive provision of NADH to the respiratory chain in a similar manner to ethanol reassimilation. Kasperski (2008) skillfully modeled the bioenergetics of *S. cerevisiae* yeast to show that the interrelation between the generation of NADH and its reoxidation to NAD^+^ determines the occurrence of several phenomena such as the Pasteur and the Crabtree effects and others (Hagman et al., 2014). The author concluded that the Crabtree effect occurs because high concentrations of glucose (>110 mg/ml) result in excessive NADH provision, which creates a redox imbalance that promotes a mixed respirofermentative metabolism despite the availability of abundant oxygen. This type of metabolism produces less ATP per mole of glucose than the complete oxidation of glucose to carbon dioxide. The NADH excess cited should also cause hyperpolarization of the mitochondrial membrane, negatively affecting by a feedback mechanism both the electron flow through the mETC and NADH reoxidation. It has been known that the lower the ATP content (because impaired respiration) the higher the rate of glycolysis in *Saccharomyces*, that is, an inverse correlation exist between glycolytic flux and intracellular ATP content (Larsson et al., 1997). Otterstedt et al. (2004) therefore generated a *S. cerevisiae* strain with reduced sugar uptake that was dependent on a chimeric hexose transporter. The strain showed a full respiratory metabolism even at high glucose levels, and switches to fermentation only when oxygen was lacking. Interestingly, the Warburg effect is characterized by enhanced aerobic glycolysis and the inhibition of mitochondrial metabolism in proliferating cells. Inhibition of mitochondrial function leads to a lower production of ATP via oxidative phosphorylation and lowers the cytosolic ATP/ADP ratio to favor enhanced glycolysis (Maldonado & Lemasters, 2014). These authors proposed that the cytosolic ATP/ADP ratio is crucial to defining whether cell metabolism is predominantly oxidative or glycolytic.

Granot et al. (2003) showed that *S. cerevisiae* cells die within a few hours when sugars are available but no additional nutrients that support growth are present. In contrast, cells incubated in water or in the presence of other nutrients but without sugar remained viable for weeks. This sugar-induced cell death was characterized by high ROS production, RNA and DNA degradation, membrane damage, nuclear fragmentation, and cell shrinkage. Since yeast cells that were deficient in hexose phosphorylation did not die, the results seem consistent with the model in which high sugar levels cause an excessive provision of NADH, which in the absence of other nutrients could not be reoxidized to NAD^+^ to maintain the redox balance. ROS are known to intervene in apoptotic execution and this link is well conserved during evolution (Madeo et al., 2004). Therefore, a redox imbalance of the NADH/NAD^+^ couple may justify the high ROS production and the cellular damage observed. This behavior is strikingly similar to the cell death observed in *P. rhodozyma* when actively assimilating ethanol under copper deficiency (Martínez-Cárdenas et al., 2018). Yeast hexose transporter genes (*hxt*) are known to be induced by different levels of glucose (Ozcan & Johnston, 1995). In particular, the induction of HXT1 depends on the availability of high concentrations of glucose hence the carbon flow through the glycolytic and TCA pathways can be regulated upstream at the transporter level. A dysfunctional HXT1 that causes a much greater carbon flow through the central metabolism is therefore expected to result in redox imbalances and oxidative stress.

Luengo et al. (2021) recently examined several different proliferating cells, to study the metabolic consequence of activating the pyruvate dehydrogenase complex (PDH), which catalyzes the oxidation of pyruvate to acetyl-CoA to increase pyruvate oxidation at the expense of lactic fermentation. Increased PDH activity was found to impair the proliferation of cells by increasing the NADH/NAD^+^ ratio. High NADH/NAD^+^ ratios cause hyperpolarization of the mitochondrial membrane, negatively affecting the electron flow in the mETC and NADH reoxidation. Paraphrasing the conclusions of the authors, it is striking that cells engage in aerobic fermentation (lactic acid production) under an excessive supply of NADH as compared to the amount that is actually needed to support the cellular ATP turnover. Accordingly, insufficient ATP demand slows-down the reoxidation of NADH and electron flow through the mETC. In contrast, the authors found that increasing ATP hydrolysis (by treatment with gramicidin D), or uncoupling respiration from ATP synthesis [by treatment with trifluoromethoxy carbonylcyanide phenylhydrazone (FCCP)] restores the NADH/NAD^+^ homeostasis and proliferation. Increasing NADH reoxidation allows proliferation, despite the increased pyruvate oxidation, merely because the oxidative stress confronted by the cells is decreased. Whenever NADH reoxidation is constrained through mitochondrial respiration, cells trigger aerobic fermentation despite the abundance of available oxygen. This allows cells the reoxidation of the excess of NADH and they can therefore avert harsh oxidative stress. In addition, the resultant increase in NAD^+^ regeneration supports a higher glycolytic flow for cell proliferation. Accordingly, it is easy to discern that the NADH/NAD^+^ pair acts as a two-way signaling couple. High NADH/NAD^+^ ratios promote ROS production and activate ROS signaling pathways, whereas a more oxidized state of the NAD^+^/NADH couple signals NAD^+^ regeneration and ATP consumption. NAD^+^ fuels oxidation reactions of the central carbon metabolism (e.g., glycolysis and TCA cycle) to allow proliferation. It should be noted that ADP, ATP and NAD^+^ are all cofactors that are required for efficient glycolysis and TCA function.

In a final example, yeast cells were forced to switch from glucose to the fatty acid oleate in a glucose-limited chemostat (Koerkamp et al., 2002). Immediately after the switch, a transient and specific stress response was noted, including the upregulation of the enzymes necessary for ROS detoxification. Oleate is a more reduced carbon substrate than glucose, meaning that its oxidation yields more reducing equivalents per carbon equivalent compared to glucose (Villadsen et al., 2011). Thus, we can surmise that oleate oxidation could provide an excess of reducing equivalents to the respiratory chain, raising either the NADH/NAD^+^ or QH_2_/Q ratio, or both, thus promoting ROS generation. Yap1 transcription factor is a major regulator of the stress response in *S. cerevisiae* (Wood et al., 2004). Upon activation by increasing levels of ROS, Yap1 rapidly redistributes to the nucleus where it regulates the expression of tens of genes. Other transcription factors involved in H_2_O_2_ sensing in bacteria, lower eukaryotes and mammalian cells have been reviewed earlier (Marinho et al., 2014). The cellular stress response is a universal mechanism that represents a defense reaction of cells to damage that environmental forces (or genetic defects) inflict on macromolecules (Kultz, 2005). In fact, many stress responses are usually coupled with arrest of growth and cell-cycle progression, which serves to reallocate cellular resources toward defense.

## Discussion and Perspectives

A number of studies have shown that accumulation of ASX by *P. rhodozyma* cells occurs under a broad range of conditions that mostly inhibit cell growth, including; several nutrient deficiencies, the presence of respiratory inhibitors, or certain distinctive gene mutations and physical agents (light). However, the molecular mechanisms that relay each stimulus to induce suitable gene expression and thereupon promote ASX synthesis remain poorly understood.

**Fig. 4:**
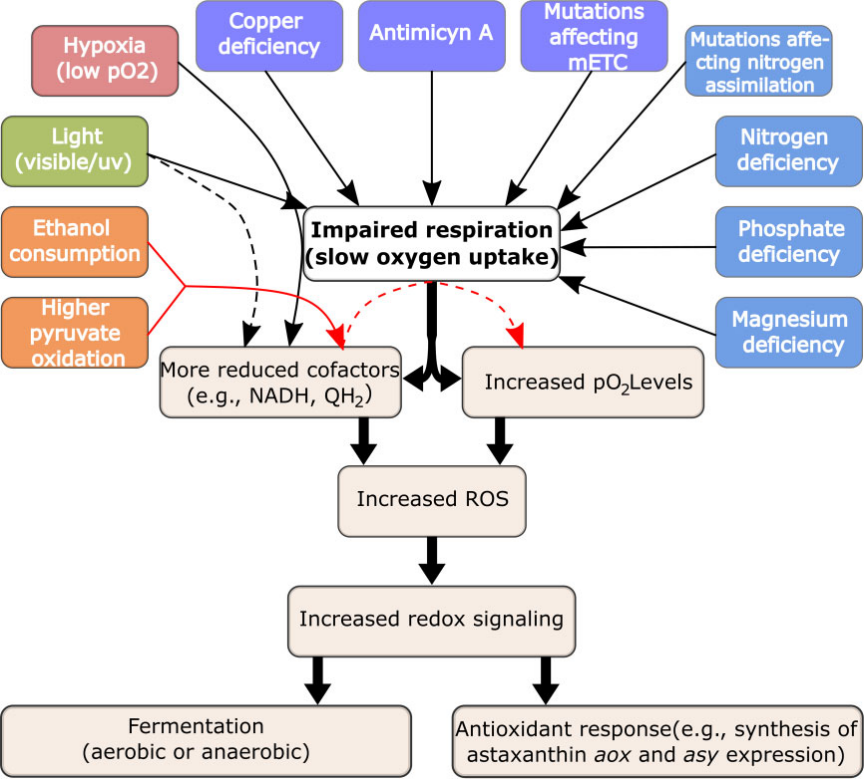
Proposed mechanism by which *P. rhodozyma* cells relay several nutrient deficiencies, the presence of respiratory inhibitors, certain distinctive genetic mutations, and physical cues (light) to trigger an adaptive response. The mechanism rationalizes the central role that is played by redox imbalances along with oxidative milieu to generate dynamic redox signals that inform the yeast cells about the changing nutritional and environmental conditions or genetic traits. The dynamic redox signal may serve to steadily tune an adaptive response. The induction of ASX-synthesis, fermentation, and *aox*-expression either requires the occurrence of a redox imbalance that is caused by conditions belonging to three groups: (i) Impairment to the electron flow through the mETC, via copper deficiency, the presence of respiratory inhibitors such as antimycin A or gene mutations (dark blue frames). (ii) Impairment to oxidative ADP-phosphorylation via N deficiency, P deficiency, the presence of inhibitors that affect the F_0_F_1_-ATP synthase function and mutations that impair N metabolism (light blue frames). (iii) The excessive provision of NADH, via the active reassimilation of ethanol or the use of molecules that increase pyruvate oxidation in other cell types (orange frames). Events (i) and (ii) lead to similar sequential outcomes: (a) The impaired reoxidation of NADH to NAD^+^, which is concurrently with impaired respiration. (b) An increase in mitochondrial pO_2_ together with an increase in the NADH/NAD^+^ ratio. (c) Higher O_2_^·−^/H_2_O_2_ formation as a result of the events (b). (d) Induction of an antioxidant response (e.g., ASX synthesis and expression of *asy* and *aox*). (e) Induction of alcoholic fermentation (low oxygen levels trigger anaerobic fermentation, whereas copper deficiency promotes aerobic fermentation). The dynamic increases of the NADH/NAD^+^ ratio and pO_2_ (cited in b) that result from impairment of the oxidative phosphorylation of ADP are not always uniform due to the variable degree of the coupling between the oxidative phosphorylation of ADP and electron flow through the mETC. Moreover, ethanol consumption at the diauxic shift increases the NADH/NAD^+^ ratio as primary outcome of an excessive NADH production. Red arrows show that the conditions (iii) lead to redox imbalances that primarily result from the excessive provision of NADH (and not from impaired respiration) followed by impairment of the respiratory chain and higher pO_2_. These events lead successively to similar outcomes as those cited above in (c) (d) and (e). The induction of fermentation, as in (e) does not occur when ethanol or some other nonfermentable carbon source is utilized but may be functional when the rapid provision of NADH comes from high glucose concentrations (Kasperski, 2008). The dashed black arrow shows the likely reduction of redox cofactors driven by light in *P. rhodozyma* that promote ROS production. The black arrow that emerges from hypoxia indicates that this condition mainly affects the NADH/NAD^+^ ratio but (normally) not the pO_2_ level. *P. rhodozyma* cells may activate an antioxidant response (i.e., the expression of *asy* and *aox*) and fermentation (when fermentable carbon sources are used).

Based on the recent analysis of our own data (Martínez-Cárdenas et al., 2018), and data from others, we proposed a model framework to highlight the fact that intracellular redox imbalances in the NADH/NAD^+^ couple in association pO_2_ are the decisive driving forces that promote cellular ASX accrual (Fig. 4). In the present work, we performed a broad review of the available data on ASX synthesis, using more data than has been examined previously. As a result, we propose that our recently proposed model (Martínez-Cárdenas et al., 2018) can also be suitable to rationalize the onset of cellular ASX accrual under a wider range of dissimilar conditions (Fig. 4). Furthermore, we have shown that activation of other relevant processes such as fermentation, regardless of whether it is under aerobic or anaerobic conditions, and *aox*-expression, exemplify the crucial role that development of redox imbalances, leading to higher reduction degree of the NADH/NAD^+^ couple, play in triggering such and possibly other processes.

We intend to highlight the fact that conditions leading to the induction of ASX synthesis, *aox-*expression and fermentation, require the development of redox imbalances caused by conditions that can be grouped as follows: (i) The impairment of electron flow through the mETC (e.g., copper deficiency, the presence of respiratory inhibitors such as antimycin A, gene mutations impairing the electron flow through the mETC). (ii) The impairment of oxidative ADP-phosphorylation (e.g., supposedly under nitrogen deficiency, phosphate deficiency, the presence of inhibitors that affect the F_0_F_1_-ATP synthase function, or mutations that impair nitrogen metabolism). (iii) Conditions that lead to an excessive provision of NADH (e.g., the active reassimilation of ethanol, high sugar concentrations).

Our ultimate aim was to propose an underlying mechanism that can explain how a broad range of dissimilar conditions converge to induce ASX synthesis and other processes in *P. rhodozyma* (Fig. 4). Regardless of the particular condition that induces ASX synthesis, fermentation or *aox*-expression, we argue that in all cases, the induction analogously occur following redox imbalances when oxygen is available. Since, mitochondrial ROS generation dynamically depends on the reduction degree of the NADH/NAD^+^ couple and the pO_2_ level, we can assume that induction of the cited processes occurs through a mechanism that involves an increase in the mitochondrial generation of O_2_^•–^/H_2_O_2_. That is, the activation of the above processes under conditions (i) and (ii) cited above, appears to be the result of a mechanism involving the impairment of respiratory activity that results in (a) an increase in the mitochondrial pO_2_, (b) an increase in the reduction state of redox cofactors (e.g., NADH and/or QH_2_), (c) an increase in ROS signaling, and (d) a selective activation of nuclear gene expression as cell adaptive response. Comparably, condition (iii) denote an excessive supply of NADH to the mETC leading successively to a higher NADH/NAD^+^ ratio and hyperpolarization of the mitochondrial membrane, pmf exerting feedback control over electron flow through the mETC, and impairing the reoxidation of NADH while increasing pO_2_. Analogously, these latter two factors may increase ROS signaling. Thus, using an analogous mechanism that depends on NADH and oxygen to jointly generate redox signals, *P. rhodozyma* cells can fine-tune their metabolism to match the availability of nutrients, the cell genetic makeup, or the presence of respiratory inhibitors of other.

In the past 20 years, the perception of the role of O_2_^•–^/H_2_O_2_ has changed from molecules that cause cellular damage to redox signals that mediate the adaptations of the cellular metabolism to changing environmental conditions (Chandel, 2014; Handy & Loscalzo, 2012). It is well established that ROS are required for subcellular and intercellular communication. In fact, there is a sizeable literature on the production and effects of ROS in biological systems both in relation to their implication in cell signaling pathways and the damage that they may cause (Winterbourn, 2008). ROS oxidatively modify cellular proteins, so, activity and function of these modified proteins may be tuned via the oxidation of ROS-sensitive groups. Indeed, redox signals are known to regulate protein–DNA interactions, DNA replication, and the stability of the genome (Hansen et al., 2006). Expression of most genes is conditional in every cell, and the regulation of gene expression is common to all cellular forms of life. Therefore, protein oxidation by modifying the redox state of numerous target intracellular proteins, and depending on its intensity and duration, may dynamically tune the gene expression or silence genes (Davies, 2001; Finkel, 2012; Handy & Loscalzo, 2012; Holmström & Finkel, 2014). Nevertheless, protein oxidation increases during periods of severe oxidative stress constitute a significant threat to cell survival.

We must consider that a substantial part of the mitochondrial NADH binds to mitochondrial proteins. In mammalian mitochondria, there are eleven distinct mitochondrial sites that differ markedly in their capacity to leak electrons to oxygen to produce O_2_^•–^ and/or H_2_O_2_ (Brand, 2016; Quinland et al., 2013). The topologies, capacities, and substrate dependencies of each site have recently been clarified. These different site-specific topologies are of paramount importance for redox signaling.

### The Mechanism that Signals Redox Imbalances in Other Cell Types

The biochemistry of intermediary metabolism is conserved from bacteria to humans, even though the regulatory mechanisms vary depending on the cell type (Mehlgarten et al., 2018; Otterstedt et al., 2004). It has long been known that the redox state of the NADH/NAD^+^ couple can be modulated *in vivo* by the respiratory activity of several cell types other than yeasts (Mayevsky & Chance, 2007; Mayevsky & Rogatsky, 2007; Noda et al., 2002). The NADH/NAD^+^ ratio increases when respiration slows down, whereas active respiration diminishes this ratio through the rapid conversion of NADH to NAD^+^ (Mayevsky & Chance, 1982; Sung et al., 2010). Dissimilar conditions have been shown to cause dynamic changes in the redox state of the NADH/NAD^+^ pair (Dai et al., 2016; Luengo et al., 2021; Schneckenburger & Koenig, 1992; Teodoro, Rolo, et al., 2013; van Hoek & Merks, 2012; Wilhelm & Hirrlinger, 2011; Wu et al., 2016). Nevertheless, the means by which divergent conditions translate into changes in the NADH/NAD^+^ ratio *in vivo* have not yet been integrated into a mechanism that can explain it. Below we provide some examples of reports concerning changes in the NADH/NAD^+^ ratio in other cell types, which suggest that the mechanism proposed in the present work (Fig. 4) may be relevant for other organisms beyond *P. rhodozyma*. However, since organisms that synthesize ASX are very few, the crucial antioxidant role that ASX plays in *P. rhodozyma* must be performed by some other antioxidant molecule in these other organisms. MnSOD (SOD2) appears as a clear possibility due to its known universal role as antioxidant protein in just about all cell types (Zou et al., 2017).

Respiratory deficient strains of *S. cerevisiae* show increased NAD(P)H fluorescence (4-fold) compared to native yeast strains (Schneckenburger & Koenig, 1992). *Candida albicans* and *Candida dubliniensis* display increased NADH fluorescence and decreased ATP levels after treatment with cyanide or antimycin A (Peña et al., 2015). Therefore, dynamic intracellular changes of the NADH/NAD^+^ redox state are common in many cell types. A high NADH/NAD^+^ ratio has also been shown to cause oxidative stress and induce multiple responses in different cell types (Hõrak, 2020).

Moreover, it is striking that similar fundamental principles are known to apply to photosynthetic systems where changes in light intensity and temperature promote imbalances between the light energy absorbed through photochemistry and the utilization of cell energy. Thus, excess light or a low temperature can generate redox signals to initiate gene transcription via the redox state of the plastoquinone pool (Escoubas et al., 1995; Hüner et al., 1998).

Many organisms from prokaryotes to higher eukaryotes respond to cold stress in a comparatively analogous way (Al-Fageeh & Smales, 2006). For example, yeasts growing at low temperatures below optimal slow down their growth and metabolism (Aguilera et al., 2007). Ballester-Tomas et al. (2015) have found an increased need of oxidation of NADH to NAD^+^ in *S. cerevisiae* grown at low temperature. Remarkably, low temperature increases the adenylate energy charge and glycolytic intermediates (Tai et al., 2007) that, alike as with nitrogen limitation in *P. rhodozyma* (cited above), suggest the involvement of an excess of ATP to promote an adaptive response. Heat shock in *Saccharomyces* species may also cause a redox imbalance that can be reversed by increased glycerol production (Paget et al., 2014).

Accordingly, the balanced supply and regeneration of reducing equivalents in the form of NADH seems crucial to any living cell. We showed that the NADH/NAD^+^ couple can be seen as a central link between the environment and adaptive cell responses, and this is presumably not only in *P. rhodozyma*. Other cell types including those of higher eukaryotes may operate analogously, as mentioned previously (Luengo et al., 2021). Therefore, the proposed model framework developed for *P. rhodozyma*, may be of help in understanding how other cells detect and mount an adaptive response when confronted with a broad set of diverse and ever-changing environmental, physical (e.g., light) or nutritional conditions, or genetic mutations that alter cells genetic traits. We predict that the proposed model framework may help to improve our understanding of many degenerative diseases (e.g., metabolic syndrome, atherosclerosis, neurodegenerative diseases, diabetes, cancer, and arthritis) and the aging process, which have all been associated with increased ROS production in evolutionarily distant eukaryotes (Dakik & Titorenko, 2016; Quijano et al., 2016). Both metabolic diseases and the aging process are characterized by high NADH/NAD^+^ ratios (Cortés-Rojo et al., 2020; Patgiri et al., 2020; Teodoro, Gomes, et al., 2013; Wu et al., 2016). Nonetheless, more detailed studies are warranted to better understand this issue, which persists as a fundamental question in biological research (Kemp et al., 2008; Lu and Thompson, 2012).

### The Close Connection Between Cellular Metabolism and Redox Balance

Both NADH and ATP are required to harness the energy released from the carbon source. These key molecules drive much of cell metabolism; therefore their production represents a major purpose of the cellular catabolism (Walsh et al., 2018; Xu et al., 2017). The relevance of these two molecules is evident because they are compulsory participants in several key reaction steps that are central in metabolism (e.g., glycolisis and TCA cycle) as well as other primary pathways in which they can assume crucial regulatory functions. Another purpose of catabolism is to retrieve, from glucose, the various carbon skeletons that are needed for biosynthesis of macromolecules. Thus, both NADH and ATP are closely connected with the utilization of carbon skeletons derived from a carbon source (Xu et al., 2017). For example, any impairment in DNA synthesis slowing down carbon utilization and growth, compulsorily signifies lower energy demands for the syntheses of new proteins and new yeast cell biomass. This presumably can result in excess of NADH and/or ATP that according to the mechanism proposed in this work, inhibits electron transfer through the mETC and increases ROS signaling. Similarly, in this study, based on the analysis of available data from *P. rhodozyma*, we explain that when sugars are available as carbon sources, in combination with impaired oxidative phosphorylation (e.g., N, P, Mg deficiencies, mutations that impair nitrogen assimilation or presumably mutations or drugs that inhibit the activity of F_0_F_1_-ATP synthase), may result in short-term increases in the NADH/NAD^+^ ratio and an excess of carbon skeletons, as both are derived from the low rate of ATP hydrolysis and/or production (Pan et al., 2020). Excess energy and intermediate metabolites available can thus be diverted to biosynthetic processes other than protein synthesis, such as the production of ASX, fatty acids and sterols, away. We need to bear in mind that the regulation of mass and energy flows through the cellular metabolic network is fundamental for the functioning of all living cells (Oliveira & Sauer, 2012). As possible, the flows that occur through the cellular network of metabolic pathways must be managed so that no intermediates build up to undesirable levels in the various cell compartments. In addition, the balanced use of metabolic intermediates and energy is a key issue, because at the same time the laws of conservation of matter and energy must be observed.

Yeast cells could rapidly adjust their metabolism in response to changing extracellular nutrient conditions. The expression of at least one quarter of all yeast genes in *S. cerevisiae* are affected by the specific growth rate, independent of the limiting nutrient (Brauer et al., 2008; Regenberg et al. 2006). These expression changes are similar to those found when cells are exposed to different types of stress (Regenberg et al. 2006). Metabolic fluxes and hence metabolic intermediaries, appear to be regulated hierarchically, for example, by changes of gene expression that adjust enzyme capacities (*V*_max_) and/or by interactions of enzymes with intermediary metabolites, ROS, ATP, products or allosteric effectors (Ewald et al., 2016; Mehlgarten et al., 2018). Several metabolic enzymes are subjected to multiple post-translational modifications (PTMs), suggesting that yeast cells can use different modifications and/or combinations of them to specifically modulate a particular enzyme activity (Tripodi et al., 2015). Glycolysis and fermentation are the pathways where phosphorylation, acetylation and ubiquitination are most frequent, while enzymes of storage carbohydrate metabolism are especially phosphorylated. Regulation of enzymes by PTMs provides one of the fastest ways for *S. cerevisiae* cells to adjust their metabolism to environmental cues (Oliveira & Sauer, 2012). Also, there is significant evidence that epigenetic modifications of chromatin depend on concentration of particular metabolites of intermediary metabolism, enabling the regulation of gene expression in consonance with metabolic state (Huang et al., 2015; Lu & Thompson, 2012). These epigenetic marks are most probably dynamic regardless if derived from nutrient or environmental cues, the presence of respiratory inhibitors, or distinctive gene mutations among others. In consonance, Plech et al. (2017) have proposed that the cell mechanism to lessen the effects of nutritional and environmental changes may overlap with the one used to lessen the effects of mutations. It is intriguing that from bacteria to humans, individual cells within isogenic populations can show significant variation in stress tolerance (Gasch et al., 2017). These authors also found significant regulatory variation in individual yeast cells, both before and after stress. Variation in the cellular network topology among cells may be responsible for this behavior. Thus, dynamic changes in the redox signaling, metabolic flows and intermediary molecule concentrations could be dependent on cell network topology, which may be affected by present and earlier nutritional, environmental and physical conditions, beyond of the cellular genetic traits. For example, the effect of mutations that cause defects in proteins can be mitigated by sequential adjustments in ATP, NADH/NAD^+^, which in turn modify redox signaling, cellular metabolic flows and metabolite concentrations. These latter changes in turn could, by covalent protein binding, modify the topology of the network through post-translational modifications (PTMs) and epigenetic marks that affect gene expression. Featherstone & Broadie (2002) have found that most single-gene mutations in *S. cerevisiae* not only affect the expression of the mutant gene, but also the expression of many other genes. This suggests the existence of connectivity between the genes in a gene expression network that allow yeast-cells to diminish the detrimental effects of particular mutations. Therefore, most mutant phenotypes are the result of changes in the expression of many genes.

The mechanism proposed in this work by which *P. rhodozyma* cells lessen the effects of nutritional, environmental or genetic changes (e.g., by gene mutations) could be applicable for other organisms as well (Fig. 4). NADH together with ATP levels to all appearances shall be pivotal to coordinate the overall cell metabolism in concert with oxygen. Nutritional or environmental challenges and even acquired genetic traits can be translated into a particular redox state via the central metabolism, whose structure is generally highly conserved from yeast to humans. It follows that the ATP/ADP and the NADH/NAD^+^ ratios are both integral indicators of the environment and the nutritional and genetic states of the cell. The redox signals associated with these ratios are the means by which warnings can be conveyed to cells in order to activate an appropriate adaptive response. It is striking, the close link between intermediary metabolism and redox balance that emerges from detailed consideration of the biochemistry and biology of the ATP/ADP and NADH/NAD^+^ couples, the two key energy currencies of cells. In summary, *P. rhodozyma* cells, and all living cells, strive repeatedly to preserve the global redox balance by inducing multiple processes. This adaptive response to a variety of stimuli serves, among other functions, to protect cells from the impact of conditions that favor the development of harsh intracellular oxidative stress, which often inhibits cell growth. In other instances similar conditions may pose a major hazard to cells survival.

### NAD^+^ Signaling

We have given particular emphasis to NADH in this study. However, its companion NAD^+^ has emerged recently as a key signaling molecule and co-substrate for enzymes, beyond its redox role (Berger et al., 2004; Chiarugi et al., 2012; Gakiere et al., 2018; Martens et al., 2018; Pollak et al., 2007). NAD^+^-dependent signaling pathways regulate fundamental processes such as transcription, DNA repair, cell cycle progression and apoptosis. NAD^+^ plays a major role as a substrate for poly (ADP-ribosyl) polymerase and NAD^+^-dependent protein deacetylases (sirtuins). The sirtuins are a highly conserved family of signaling enzymes that consume one molecule of NAD^+^ for every deacetylated lysine side chain. Their requirement for NAD^+^ may render them prone to regulation by shifts in NAD^+^ (Bedalov et al., 2003; Teodoro, Rolo, et al., 2013; Wierman & Smith, 2014). NAD^+^ deficiency has been found to be associated with several diseases for which intervention to increase NAD^+^ levels has been proven beneficial. Furthermore, maintenance of the mitochondrial NAD^+^ pool is also of significant importance in living organisms (Stein & Imai, 2012). A subtle balance between NAD^+^ production and degradation also regulates the cellular NAD^+^ levels (Yaku et al., 2018). Maintaining an optimal NADH/NAD^+^ ratio is essential for cellular function, as changes in the NADH/NAD^+^ redox state can largely reconfigure cell metabolism. As such, the NADH/NAD^+^ couple must be regarded as a two-way dynamic hub for cell signaling and the coordination of cellular metabolism that can relay nutritional and environmental cues, and cell genetic traits whether inherited or acquired, via redox and non-redox signals. The properties of these signals appear to be universal among all living cells. As argued, ASX synthesis can be regarded as a specific response only among the multiple cellular processes that are supposedly similarly activated according to the redox state of the NADH/NAD^+^ pair. To conclude, the question of whether ‘life is redox driven’ from the state of NADH/NAD^+^ pair, most probably will grant us a positive answer. Although *P. rhodozyma*/*X. dendrorhous* has been studied by numerous researchers for more than 45 years, there are still great prospects for this yeast either from both the fundamental and applied research points of view.
